# Interplay between microRNAs and TGF-β signaling in T cells: implications for Th9 differentiation and immune pathogenesis

**DOI:** 10.3389/fimmu.2026.1828634

**Published:** 2026-05-01

**Authors:** Tae Sung Kim, Yun-Ji Lim, WanJun Chen

**Affiliations:** 1 Department of Oral Microbiology, School of Dentistry, Dental and Life Science Institute, Pusan National University, Yangsan, Republic of Korea; 2 Education and Research Team for Life Science on Dentistry, Pusan National University, Yangsan, Republic of Korea; 3 Department of Biochemistry, School of Medicine, Pusan National University, Yangsan, Republic of Korea; 4 Research Institute for Convergence of Biomedical Science and Technology, Pusan National University Yangsan Hospital, Yangsan, Republic of Korea; 5 Mucosal Immunology Section, National Institute of Dental and Craniofacial Research, National Institutes of Health, Bethesda, MD, United States

**Keywords:** autoimmune disease (AD), cancer, CD4+ T cell, microRNA, TGF-b signalling, Th9

## Abstract

Since the discovery of microRNAs (miRNAs) in 1993, they have been established as key post-transcriptional regulators of immune cell function. In particular, miRNAs have emerged as key modulators of CD4^+^ T cell activation and differentiation. Accumulating evidence indicates that miRNAs influence CD4^+^ T cell fate through modulating transforming growth factor β (TGF-β) signaling, which negatively regulates T helper (Th) 1 and Th2 cell differentiation, while promoting the generation of regulatory T (Treg) cells, Th17 as well as Th9 cells. Despite these advances, our understanding of specific roles of miRNAs and their target molecules in CD4^+^ T cells remains limited, particularly in Th9 cells. In this review, we discuss the regulatory networks of miRNAs targeting the TGF-β signaling pathway in CD4^+^ T cells, with a special focus on their roles in Th9 cell differentiation and function.

## 1 Introduction

T lymphocytes (T cells) are the major cells of the adaptive immune system orchestrating immune responses in inflammation, autoimmunity, allergy/asthma, transplantation, cancer and infectious diseases. T cell development is initiated from CD4^-^ CD8^-^ double negative (DN) thymocytes to the CD4^+^ CD8^+^ double positive (DP) thymocytes, and eventually differentiated into CD4^+^ or CD8^+^ single positive (SP) cells (1). T cell subsets include CD4^+^ T helper (Th) cells (Th 1, Th2, Th9, Th17, Th22, Tregs and T follicular helper), and T cytotoxic (Tc) CD8^+^ cells (1). T cells are defined by surface molecules, cytokines and key transcription factors that control their differentiation (1–3).

miRNAs play crucial roles in regulating adaptive immune response mediated by T cells. Numerous studies have demonstrated that miRNAs exert pleiotropic regulatory effects on T cell differentiation and activation by fine-tuning cytokine expression and orchestrating transcriptional and signaling networks (4–8). Importantly, miRNAs are now recognized as key post-transcriptional regulators of immune cell function, and their role in shaping CD4^+^ T-cell lineage commitment and effector specialization has become increasingly evident.

Among the various Th subsets, Th9 cells have attracted substantial scientific interest due to their diverse roles in immune regulation across multiple disease settings, including tumor immunity, promotion of allergic inflammation, and modulation of immune responses during infection (9, 10). Th9 cells, primarily characterized by the production of interleukin-9 (IL-9), are differentiated from naïve CD4^+^ T cells by TGF-β in the presence of IL-4 signaling, which activates key signaling pathways including Smad2/3, STAT6, and downstream transcriptional regulators including interferon regulatory factor (IRF) 4, GATA binding protein (GATA) 3, basic leucine zipper ATF-like transcription factor (BATF), GITR, and PU.1 (*Spi1*) (9–12). This TGF-β-dependent architecture makes Th9 cells especially sensitive to miRNA-mediated modulation. These transcriptional networks, comprising IRF4, GATA3, BATF, GITR, and PU.1, cooperatively drive *Il9* gene expression and define Th9 lineage commitment.

However, research on miRNAs related to regulation of IL-9 production and Th9 differentiation has been relatively limited compared to other T cell subsets. Therefore, this review focuses on the current understanding of how miRNAs participate in the differentiation and activation of T cell subsets through TGF-β signaling, with a particular emphasis on the miRNA-mediated regulation of Th9 cell differentiation and function in the disease context.

## 2 miRNA biogenesis

miRNAs are small (~19–24 nucleotides) non-coding RNA molecules that regulate gene expression as post-transcriptional modulators (13–15). In the canonical biogenesis pathway, transcription by RNA polymerase II produces primary miRNA (pri miRNA), which is subsequently processed into precursors of miRNAs (pre-miRNA) by the RNases III enzyme Drosha/DGCR8 in the nucleus (13–15). The pre-miRNA in the nucleus is approximately ~70 nt in length, which then forms a ~22 nt miRNA-miRNA duplex by an RNase III Dicer in the cytoplasm (13–15). The single-strand mature miRNA from miRNA-miRNA duplex can bind to complementary sequences on target messenger RNAs (mRNAs) and silence gene expression by degrading mRNAs or through translational repression (13–15). Moreover, miRNAs can be secreted into extracellular fluids and transported to target cells via exosome or microvesicles (MVs) to mediate cell-cell communication (13, 16). Currently, in release 22 of miRBase, human miRNAs are represented by approximately 2,600 mature sequences in the full dataset (17), and they have the potential to modulate approximately 60% of protein-coding genes in the total human genome (18).

## 3 Functional roles and clinical relevance of miRNAs

Beyond their biogenesis, miRNAs serve as critical regulators of gene expression in immune cells, particularly in CD4^+^ T cells, by modulating mRNA stability and translation (19, 20). In these cells, miRNAs fine-tune signaling pathways and transcriptional programs that govern T cell activation and lineage commitment (4, 21). Specifically, miRNAs regulate the balance of T cell subsets (20, 22), including Th9 cell differentiation and IL-9 production, partly through modulation of the TGF-β signaling pathway. Consequently, the dysregulation of these miRNA-mediated regulatory networks can disrupt TGF-β signaling and T cell homeostasis, thereby contributing to the pathogenesis of immune-related diseases such as cancer, autoimmune disorders, and allergic inflammation, where Th9 cells exert context-dependent effects.

## 4 TGF-β signaling

TGF-β (TGF-β1, TGF-β2, and TGF-β3) belongs to TGF-β superfamily (23), and plays an important role in the regulation of the development, activation, proliferation, and differentiation of immune cells (11). TGF-β ligands bind to heterometric type II receptor (TGFβRII) and type I receptor (TGFβRI). Type III receptor (TGFβRIII; also known as betaglycan) functions as a co-receptor that facilitates the binding of TGF-β ligands to TGFβRII.

In the canonical pathway triggered by these ligands, type I receptors phosphorylate the receptor-regulated Smads (R-Smads). Typically, TGF-β isoforms can phosphorylate Smad2 and Smad3 (Smad2/3), which subsequently forms a complex with the common-partner Smad4 to accumulate in the nucleus and regulate the transcription of target genes (11, 24). In the context of CD4^+^ T cells, this complex directly or indirectly modulates the expression of key genes required for Th9 differentiation, such as the signature cytokine *Il9* and the critical transcription factor PU.1 (9, 11, 25). TGF-β can also signal through the Smad-independent non-canonical pathways, including tumor necrosis factor (TNF) receptor-associated factor 6 (TRAF6)/TGF-β-activated kinase 1 (TAK1) pathways, the phosphatidylinositol 3 kinase (PI3K)/AKT/mammalian target of rapamycin (mTOR) pathways, and Rho-like GTPases pathways (26).

Additionally, these TGF-β signaling pathways are negatively regulated by inhibitory Smads (I-Smads) including Smad7, which could be transcriptionally induced by TGF-β signaling and many other cytokines including TNF-α and INF-γ (27, 28). I-Smads, such as Smad7 can bind to DNA via their MH2 domain and disrupt Smad-DNA complex formation, thereby preventing phosphorylation and nuclear translocation of R-Smads (27, 28).

Among CD4^+^ T cell subsets, TGF-β signaling plays a critical role in Th9 cell differentiation. In the presence of IL-4, TGF-β drives the differentiation of naïve CD4^+^ T cells into Th9 cells by activating Smad2/3-dependent transcriptional programs (9, 29). In addition, TGF-β-mediated suppression of alternative lineage-specifying pathways further facilitates Th9 lineage commitment. This highlights TGF-β signaling as a central molecular axis linking extracellular cues to Th9 differentiation and function, thereby providing a mechanistic foundation for the miRNA-mediated regulatory networks discussed in the following sections.

## 5 miRNAs and TGF-β signaling during T cell differentiation

The regulatory interplay between miRNAs and TGF-β signaling during CD4^+^ T cell differentiation is highly dependent on both spatial and temporal contexts (30, 31). Spatially, T cell development is initiated in the thymus, where TGF-β contributes to early lineage decisions and central tolerance (32, 33). However, the majority of miRNA-mediated regulation of TGF-β signaling in CD4^+^ T cells occurs in secondary lymphoid organs such as lymph nodes and spleen, where naïve T cells are activated and differentiate into effector subsets, including Th9 cells (30). In peripheral tissues, particularly under inflammatory or disease conditions, miRNAs further modulate TGF-β signaling to fine-tune effector functions, plasticity, and cytokine production of differentiated T cells (34).

Importantly, the expression and functional impact of specific miRNAs are not uniform across these anatomical compartments. Many of the studies described below are derived from context-specific settings, including *in vitro* differentiation systems, disease models, or patient-derived samples. Therefore, the reported miRNA and TGF-β interactions should be interpreted within their respective biological contexts, as their regulatory roles may differ depending on the site of T cell activation and function.

Temporally, TGF-β signaling exerts stage-specific effects during T cell differentiation (35, 36). Early TGF-β signaling is critical for lineage commitment, including the induction of transcriptional programs required for Th9 differentiation (9), whereas sustained or late-phase signaling contributes to the maintenance, stability, and functional specialization of effector T cells (33, 37). miRNAs are dynamically regulated throughout these stages and act as fine-tuners of TGF-β signaling outputs in a time-dependent manner (19, 30). Thus, the timing of miRNA expression relative to TGF-β exposure and co-stimulatory cues acts as a decisive factor controlling T cell fate decisions.

### 5.1 miRNAs regulate TGF-β signaling in T cells

In this section, we summarize studies performed in diverse experimental systems, including *in vitro* differentiated T cells, animal models, and human disease samples. Where applicable, we highlight the specific biological context (e.g., tissue localization and disease condition) in which these miRNAs and TGF-β interactions have been reported.

There have been numerous studies that TGF-β signaling pathway is modulated by miRNAs, exerting a strong impact on the production of cytokines and transcription factors in T cells (4, 7). Although most studies have focused on Treg and Th17 cells, these regulatory mechanisms are highly relevant to Th9 differentiation, as Th9 cells also depend on TGF-β signaling in a context-dependent manner.

Severin et al. (38) revealed that the expression of several miRNAs, including miR-141 and miR-500a, target TGFβRI and Smad4 to suppress Treg activation in human CD4^+^ T cells. let-7i also inhibits Treg differentiation in patients with multiple sclerosis (MS), which is attributed to the reduction of TGFβRI and insulin-like growth factor 1 receptor (IGF-1R) on naive CD4^+^ T cells (39). miR-17 in CD4^+^ T cells can inhibit TGF-β signaling through targeting TGFβRII, promoting Th1 cell proliferation while inhibiting Tregs (40, 41). In CD4^+^ and CD8^+^ T cells from patients with MS, the significantly upregulated miR-142 targets TGFβRI and TGFβRII to suppress protective genes such as suppressor of cytokine signaling 1 (SOCS1), therefore this phenomenon can lead to the stabilizing of Foxp3^+^ Tregs while the inhibiting of conversion of IFN-γ-producing Th1 differentiation (42).

Glycoprotein A repetitions predominant (GARP) is a transmembrane protein for binding and activating latent TGF-β, thus promoting secretion and activation of TGF-β. It is found that GARP is expressed highly on the cell surface of activated Tregs but not on other Th cells. To control these processes, some miRNAs target the GARP3’ UTR. Several miRNAs including miR-142-3p, miR-185, miR-181a, miR-181b, miR-181c and miR-181d play roles in the downregulation of GARP and suppression of latent TGF-β1 secretion in CD4^+^ CD25^+^ T cells (43, 44), consequently these 6 miRNAs exhibit lower expression in human CD4^+^ CD25^+^ Tregs than CD4^+^ CD25^-^ T cells (43).

Moreover, miR-200c and miR-141 affect the downregulation of zinc finger E-box binding homeobox (ZEB), TGFβRIII and TGF-β1-mediated Smad2/3 pathway in human T cell prolymphocytic leukemia (45–47). In the overexpression of miR-128/miR-212/miR-708 combination, Smad2/3 signaling is defective in CD4^+^ T cells, leading to disrupt the development of Tregs and exacerbate the risk of central nervous system (CNS) autoimmunity in MS (48). The downregulation of miR-145 impairs the functions of T cells by targeting Smad3 in acute lymphoblastic leukemia (ALL) patients (49). Meanwhile, the upregulation of miR-181a in Tregs could promote TGF-β1/Smad signaling activation, and affect the activity of Treg cells in acute gouty arthritis (AGA) mouse models (50). Similarly, miR-181c enhances TGF-β-induced Smad2/3 signaling by targeting Smad7, which promotes Th17 cell differentiation (51). Furthermore, miR-21 also targets Smad7, thus promotes Th17 cell differentiation (52), while negatively regulates Tregs (53). In a same context, loss of TGFβRII-Smad signaling in T cells leads to increased expression of miR-21, resulting in upregulation of pro-inflammatory cytokines such as IFN-γ and TNF-α, thereby contributing to the maintenance of the effector phase of T cells (54).

miR-182 is contributed to the increased production of TGF-β, IL-6, IL-17, and Foxp3 toward the transitional state of Th17 and Tregs, respectively, although it inhibits forkhead box O1(FOXO1), T cell receptor (TCR)/CD3 complex, nuclear factor of activated T cells (NFAT), and IL-2 signaling pathways (55).

A recent study revealed that miR-126 silencing could reduce Foxp3^+^ Tregs, which is accompanied by decreased expression of TGF-β, IL-10, cytotoxic T lymphocyte-associated antigen 4 (CTLA-4) and glucocorticoid-induced TNFR-related gene (GITR), and impair the suppressive function of Tregs (56). On the other hand, miR-30a reduces the suppressive capacity of CD4^+^ inducible Treg cells (iTregs) by regulating SOCS1 in tumor environment *in vivo* (57). During this process, the overexpression of miR-30a in iTregs significantly reduces TGF-β, IL-10, CTLA-4 and GITR, which is consistent with the reduced suppressive activity of iTregs (57). miR-106b negatively regulates TGF-β and Foxp3^+^ Treg differentiation via targeting nuclear receptor subfamily 4 group A member 3 (NR4A3), thus induces immune imbalance of Treg/Th17 during the development of immune thrombocytopenic purpura (ITP) (58). Intriguingly, miR-200a is suggested as a critical player in inducing Th17 while suppressing Tregs and TGF-β in CD4^+^ T cells from psoriasis patients (59). Additionally, miR-466a targets TGF-β2 to decrease Foxp3^+^ Treg cell generation in mouse CD4^+^ T cells (60). As TGF-β signaling can affect many cell types at distance, its regulation by miRNAs should be important for the maintenance of homeostasis and the acquisition of function of T cells including Tregs.

Collectively, these studies reveal that miRNAs regulate TGF-β signaling in a coordinated manner to control CD4^+^ T cell lineage decisions. A recurring pattern is that several miRNAs, such as miR-21 and miR-181 family members, enhance TGF-β/Smad signaling by targeting inhibitory molecules like Smad7, thereby promoting Th17 differentiation while modulating Treg stability. In contrast, other miRNAs, including miR-17 and miR-106b, suppress TGF-β receptor expression or downstream signaling components, leading to reduced Treg differentiation and enhanced effector T cell responses. Importantly, these regulatory axes collectively shape the cytokine and transcriptional environment that may influence Th9 differentiation, particularly under conditions where TGF-β signaling is finely tuned in the presence of IL-4.

Notably, several recurring regulatory patterns emerge across these findings. miRNAs such as miR-128/212/708, miR-145, miR-21 and members of the miR-181 family consistently enhance TGF-β-Smad2/3 signaling by suppressing inhibitory molecules like Smad7, thereby favoring differentiation toward Th17 and, potentially, Th9 lineages. In contrast, miRNAs that target TGF-β receptors (e.g., miR-500a, let-7i, miR-17, miR-141, miR-142, miR-200c) form a functionally coherent group that dampens upstream TGF-β sensitivity and generally antagonizes Treg differentiation. A third pattern is observed in the GARP-targeting miRNAs (e.g., miR-142-3p, miR-181 family, miR-185), which converge on the regulation of latent TGF-β activation. Together, these shared mechanisms reveal that miRNA groups, rather than isolated individual miRNAs, coordinately modulate the overall amplitude and quality of TGF-β signaling.

To facilitate a clearer overview of the studies discussed above, the key miRNAs, their molecular targets within the TGF-β signaling pathway, and their functional roles in T cell regulation are summarized in **Table 1**. In addition, the table highlights whether each study was conducted under physiological or pathological conditions, providing further context for the interpretation of these findings.

**Table 1:** miRNA-mediated regulation of TGF-β signaling in T cells.

miRNA	Target(s)	Effect on TGF-β signaling	T cell subset/function	Biological context
miR-500a	TGFβRI, Smad4	Inhibition	Treg ↓	Physiological (human CD4^+^ T cells)
let-7i	TGFβRI, IGF-1R	Inhibition	Treg ↓	Pathological (MS)
miR-17	TGFβRII	Inhibition	Th1 ↑/Treg ↓	Physiological
miR-142	TGFβRI, TGFβRII	Inhibition	Th1 ↓/Treg stabilization	Pathological (MS patients)
miR-141	TGFβRI, Smad4	Inhibition	Treg ↓	Physiological (human CD4^+^ T cells)
	ZEB, TGFβRIII	Inhibition	T cell dysfunction	Pathological (Leukemia)
miR-200c	ZEB, TGFβRIII	Inhibition	T cell dysfunction	Pathological (Leukemia)
miR-128/212/708	Smad2/3	Inhibition	Treg ↓	Pathological (CNS)
miR-145	Smad3	Inhibition	T cell dysfunction	Pathological (ALL)
miR-181a	Smad signaling	Activation	Treg ↑	Pathological (AGA)
miR-181c	Smad7	Activation	Th17 ↑	Physiological
miR-21	Smad7	Activation	Th17 ↑/Treg ↓	Physiological (Multiple diseases)
miR-466a	TGF-β2	Inhibition	Treg ↓	Physiological
miR-142-3p	GARP	Inhibition	Treg ↓	Physiological
miR-181 family	GARP	Inhibition	Treg ↓	Physiological
miR-185	GARP	Inhibition	Treg ↓	Physiological
miR-30a	SOCS1	Inhibition	iTreg ↓	Pathological (Tumor microenvironment)
miR-106b	NR4A3	Inhibition	Th17 ↑/Treg ↓	Pathological (ITP)
miR-182	FOXO1, NFAT	Modulation	Th17/Treg transition	Physiological
miR-126	Indirect (TGF-β, IL-10, CTLA-4, GITR ↓)	Inhibition	Treg ↓	Pathological
miR-200a	Indirect (TGF-β)	Inhibition	Th17 ↑/Treg ↓	Pathological (Psoriasis)

Studies were categorized based on experimental settings, including *in vitro* systems, animal models, and human disease samples.

increase (↑) or decrease (↓) in T cell differentiation or frequency.

### 5.2 TGF-β signaling regulates miRNAs expression in T cells

TGF-β signaling also regulates the expression of miRNAs to control T cell differentiation. TGF-β stimulation can induce miR-10a, which limits the expression of Bcl-6 and Ncor2 to promote the stability of Tregs while inhibiting Th17 cell differentiation (61). Additionally, TGF-β signaling has been shown to regulate miRNA networks through modulation of Lin28b expression. Naïve fetal CD4^+^ T cells from human fetal secondary lymphoid organ exhibit highly expressed Lin28b that is promoted by TGF-β signaling (62). Lin28b is well known as a suppressor for let-7 family miRNAs, thereby influencing T cell differentiation programs (62). The regulatory axis is also associated with expressions of TGFβRI, TGFβRIII, and Smad2, and it further contributes to the differentiation of naïve fetal T cells into Foxp3^+^ CD25^+^ Tregs (62). miR-155 is induced in TGF-β-stimulated T cells resulting in suppression of IL-2 production and impairing T cell activation in TCR-activated CD4^+^ T cells (63). In human γδ T cells, miR-181a is upregulated by TGF-β stimulation and impairs the expression of Notch2 and Map3k2, thereby ultimately restrain γδ T cell differentiation (64). In CD4^+^ T cells from psoriasis patients and mouse models, TGF-β and IL-23 enhance miR-210 expression by inducing hypoxia-inducible factor (HIF)-1α (65). During this process, miR-210 in regulating T cells targets signal transducer and activator of transcription (STAT) 6 and Lyn, which contributes to the imbalance by inducing Th1 and Th17 cells but inhibiting Th2 differentiation. Yuting et al. (66) found that miR-99a expression is induced by TGF-β, and suppress glycolysis in CD4^+^ T cells, thereby inhibiting Th1 cell and promoting Tregs differentiation.

However, Ichiyama et al. (67) identified that Dicer1-regulated miR-183c is expressed by IL-6 signaling during Th17 cell differentiation, and this mRNA is strongly suppressed by TGF-β. TGF-β-mediated repression of miR-183c induction inhibits the pathogenic function of Th17 cells, thereby ameliorating experimental autoimmune encephalomyelitis (EAE). Consistent with this paper, TGF-β also suppresses miR-221 and miR-222 in Th17 cells (68). Both miRNAs are negative feedback regulators downstream of IL-23 to modulate the magnitude of proinflammatory Th17 response and to protect against Dextran sulfate sodium (DSS)-induced mucosal damage.

Taken together, TGF-β signaling dynamically regulates miRNA expression to fine-tune T cell differentiation programs. These miRNAs often function as feedback regulators that either reinforce or attenuate TGF-β-driven signaling pathways, thereby shaping the balance between regulatory and inflammatory T cell subsets. Given that Th9 differentiation critically depends on the strength and context of TGF-β signaling, such miRNA-mediated feedback loops are likely to play an important role in establishing the transcriptional and cytokine milieu that supports Th9 cell development and function.

Importantly, these spatial and temporal patterns of miRNA regulation provide a conceptual framework for understanding how TGF-β responsiveness shapes Th9 differentiation. miRNAs that amplify Smad2/3 signaling may create a more permissive environment for IL-4-driven Th9 lineage induction, whereas miRNAs that suppress TGF-β receptors or dampen latent TGF-β release may restrict Th9 commitment. Thus, the miRNA-TGF-β network outlined in Sections 5.1 and 5.2 establishes a mechanistic foundation for the Th9-centered regulatory axes discussed in later sections.

**Figure 1:**
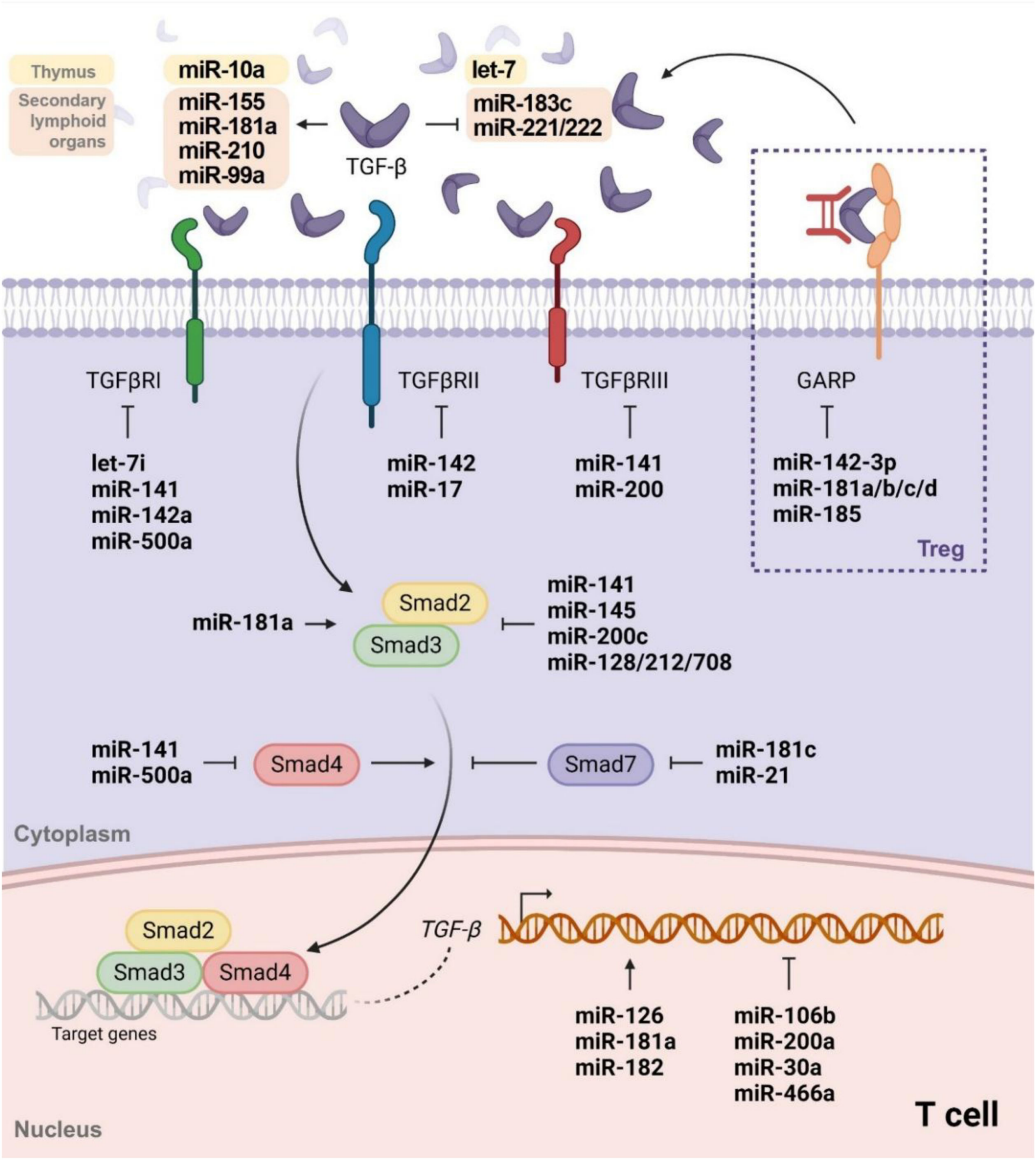
Overview of miRNAs and TGF-β signaling during CD4^+^ T cell differentiation. This figure summarizes miRNAs induced by TGF-β stimulation and the miRNAs regulating targets of TGF-β signaling cascade. The detailed subset specific effects of miRNAs on CD4^+^ T cell differentiation are further summarized in **Tables 1**, **2**.

The key findings regarding TGF-β-mediated regulation of miRNA expression and their molecular targets are summarized in **Table 2**; **Figure 1**. All these findings collectively enhance our understanding of miRNAs function in TGF-β-mediated T cell differentiation.

**Table 2:** TGF-β-mediated regulation of miRNA expression in T cells.

miRNA	Target(s)/Pathway	Regulation by TGF-β	T cell subset/function	Biological context
miR-10a	Bcl-6, Ncor2	Induced	Th17 ↓/Treg stabilization	Physiological
miR-155	IL-2	Induced	T cell activation ↓	Physiological
miR-181a	Notch2, Map3k2	Induced	γδ T ↓	Physiological
miR-210	STAT6, Lyn	Induced (+IL-23)	Th1 ↑/Th17 ↑/Th2 ↓	Pathological (Psoriasis)
miR-99a	Glycolysis	Induced	Th1 ↓/Treg ↑	Physiological
let-7 family	Differentiation regulators	Suppressed (via Lin28b)	Treg ↑	Physiological (fetal)
miR-183c	IL-6 pathway	Suppressed	pathogenic Th17 ↓	Pathological (EAE)
miR-221	IL-23 pathway	Suppressed	Th17 modulation	Pathological (DSS colitis)
miR-222	IL-23 pathway	Suppressed	Th17 modulation	Pathological (DSS colitis)

The table summarizes the miRNAs regulated by TGF-β signaling, including their molecular targets, downstream pathways, and functional impact on T cell differentiation. The data are derived from diverse experimental settings, including *in vitro* T cell differentiation systems, animal models, and human disease samples, reflecting the context-dependent regulation of miRNA expression by TGF-β.

increase (↑) or decrease (↓) in T cell differentiation or frequency.

## 6 miRNAs regulate Th9 cells

To differentiate into Th9 from naïve CD4^+^ T cells, TGF-β stimulation in conjunction with IL-4, is necessary (11, 69, 70). Among them, Th9 cells differentiation is known to be strongly triggered by stimulation of IL-4 and TGF-β (11) or IL-4 and IL-1β (71) from naïve CD4^+^ T cells, which is mainly characterized by the secretion of IL-9 and IL-10 (10, 72). In addition, downstream of TGF-β signaling, Smad2/3 and Smad4, are also required for this process (25, 29, 73). Some other transcriptional factors, including STAT6, IRF4, GATA3, BATF, GITR, and PU.1, are also reported to be required for Th9 differentiation (10). Notably, TGF-β1 and IL-4 downregulate Inhibitor of DNA binding 3 (ID3) expression in a TAK1-dependent manner. As ID3 functions as a negative regulator of Th9 development, its downregulation promotes the binding of E2A and GATA-3 to the *Il9* promoter, thereby enhancing *Il9* transcription (73).

As a diverse repertoire of miRNAs have been reported to participate in the TGF-β signaling pathway, and TGF-β signaling plays a critical regulatory role in Th9 cell differentiation, it is plausible that these previously identified miRNAs, as well as other yet unidentified ones, may also influence Th9 cell development and function. While many miRNAs influence general T cell biology, only a subset directly intersects with the TGF-β-Smad-IL-9 axis that defines Th9 lineage commitment. Therefore, in this section, we focus specifically on miRNAs that modulate TGF-β signaling components or Th9-associated transcriptional programs. Indeed, we and others have provided compelling evidence demonstrating that various miRNAs can differentially regulate Th9 cells. We thus aim to discuss the roles of miRNAs in Th9 differentiation in this section.

Given that Th9 cell differentiation critically depends on TGF-β signaling, it is essential to understand how miRNAs regulate this pathway in the context of Th9 development. While many miRNAs have been reported to influence general T cell differentiation, only a subset has been directly linked to the TGF-β-Th9 axis. In this section, we focus on miRNAs that either directly regulate TGF-β signaling components or modulate downstream transcriptional programs associated with Th9 differentiation and IL-9 production.

### 6.1 miRNA-145

Recently, Huang et al. (74) identified that miR-145 can suppress Th9 differentiation and IL-9 production by inhibiting the activation of PI3K/Akt/mTOR/p70 ribosomal protein S6 kinase (p70S6K)/HIF-1α signaling pathway in malignant ascites from liver cancer. Additionally, it was demonstrated that nuclear factor of activated T cells 1 (NFATc1) is another direct target of miR-143/145, and its modulation by miRNA negatively impacts Th9 differentiation (75). NFATc1 is an important regulator of the development, differentiation, tolerance and apoptosis of T cells (76, 77). It is well established that as a transcription factor, NFATc1 is required for numerous T cell differentiation-related genes, including *Il2*, *Il4*, *Il6*, *Il10*, *Il12*, *Il17*, *Il21*, *Tbx21*, *Gata3*, *Retinoic acid-related orphan receptor(ROR)γt*, *Cd25* and *Foxp3* (76). NFATc1 can also bind to the *Il9* promoter region and initiate transcription in cooperation with nuclear factor kappa B (NF-κB) in CD4^+^ T cells, thus playing a role in the regulation of *Il9* expression (78). Because HIF-1α and NFATc1 act downstream of TGF-β-driven transcriptional and metabolic programs, miR-145 exerts a restrictive influence on the TGF-β-Th9 axis by dampening pathways necessary for optimal IL-9 induction.

### 6.2 miRNA-146a

miR-146a is highly expressed upon T cell activation in mouse and human, and is linked with the generation of various T cell subsets (4). Particularly it inhibits IFN-γ-producing Th1 differentiation by the downregulation of its common targets, STAT1 (79) and STAT4 (80). Indeed, the increased miR-146a by Tregs restrains the production of IFN-γ and prevents Th1-mediated immunopathology (81). T cell-intrinsic miR-146a is also required to regulate a polarized Th2 response via reducing target genes i.e., STAT1, Itch and Smad4, which may contribute to the decreased inflammation in house dust mite (HDM)-challenged allergy (82). Furthermore, the deficiency of miR-146a in T cells leads to a mixed Th1 and Th17 response in otherwise Th2-dominated environments, suggesting that miR-146a regulates the differentiation of polarized cells (82). However, there are no reports on the function and targets of miR-146a in Th9 cells.

While miR-146a is increased by PU.1 in response to TGF-β (83, 84), this miRNA also reduces PU.1 (85). As mentioned above, PU.1 is an important transcription factor of *Il9* in response to TGF-β signaling during Th9 development (86). Likewise, miR-146a negatively regulates the activation of NF-κB in CD4^+^ and CD8^+^ T cells (87). Since NF-κB activity is also a crucial signaling event for *Il9* expression (71), it is possible that miR-146a might contribute directly or indirectly to the regulation of Th9 differentiation by targeting PU.1 and NF-κB, two essential regulators of IL-9 expression.

### 6.3 miRNA-15b/16

Two miRNAs, miR-15b and miR-16, have been implicated to restrict the cell cycle, survival, and memory cell differentiation of T and B cells (88, 89). miR-15b/16 is recognized as a critical inducer of Treg cells through the downregulation of mTOR signaling pathway (90). However, a recent report showed that the activation of miR-15/16 cluster restricts effector Treg differentiation by targeting interferon regulatory factor (IRF) 4 (91). Furthermore, Liu et al. (92) suggested that miR-15b in CD4^+^ T cells negatively regulate Th17 differentiation by reducing the transcriptional activity of NF-κB, thereby inhibiting RORγt gene expression.

However, there is limited evidence on the requirements for miR-15b/16 in the development and differentiation of Th9 cells. Singh et al. (93) reported that miR-15b/16 expression suppresses IL-9 production by targeting HIF-2α during Th9 differentiation, while simultaneously enhances Foxp3^+^ iTregs. Because HIF-2α supports IL-9 expression downstream of TGF-β, miR-15b/16 functions as a negative regulator within the TGF-β-Th9 axis, selectively regulating only Th9 and iTreg differentiation, but not other subsets such as Th1, Th2 or Th17 cells (93).

### 6.4 miRNA-155

Previous studies have shown that miR-155 is increased among CD4^+^ T cell subsets, and has multiple target genes for regulating T cell differentiation during their activation (94–96), suggesting functional importance of this miRNA in T cells. Specifically, miR-155 is described as a key regulator of Th2-driven immunity (82, 97, 98). Malmhäll et al. (99) described an increased IL-4-producing Th2 in miR-155 knockout CD4^+^ T cells. Meanwhile, IFN-γ receptor alpha chain (IFN-γRα) is decreased by miR-155 in activated CD4^+^ T cells, and this promotes Th1 differentiation by decreasing sensitivity to the anti-proliferative effects of IFN-γ (100). The expression of miR-155 in T cells is necessary for proper Th17 cells in airway allergy (98), EAE (101) and DSS-induced colitis (102). In the absence of miR-155, Th17 cells can elevate transcripts of DNA-binding protein *Jarid2*, coinciding with the failure to express *Il22*, *Il10*, *Il9* and *Atf3* (95).

For Th9 cells, it has been reported that CD4^+^ T cells isolated from bronchoalveolar lavage fluid (BALF) of patients with methicillin-resistant *Staphylococcus aureus* (MRSA) pneumonia, overexpression of miR-155 significantly upregulates IL-9 secretion and Th9 cells differentiation by targeting SIRT1 (103). To add the complexity about the relationship between miR-155 and Th9 cell development, it is reported that the transcription factor PU.1 is negatively regulated by miR-155 (85). Importantly, TGF-β directly induces miR-155 expression, placing miR-155 firmly within the TGF-β-miRNA-Th9 circuit and supporting its role as a positive regulator of IL-9-producing Th9 cells.

### 6.5 miRNA-23-27–24

Based on the role of miRNAs in T cell immunity, miR-24 is involved in the differentiation of Th1, Th17 and iTregs via targeting Smad7 (104). In contrast, miR-23 and miR-27 could limit Th17 and iTregs (104, 105). Furthermore, these perturb Treg cell function and therefore blocks tolerance induction (105). In this regard, considering that miR-23-27–24 cluster miRNAs (miR-23b, miR-27b, and miR-24-1) target Smad3, Smad4 and Smad5 (106), the regulation of TGF-β signaling pathway by these miRNAs is likely to be crucial in the modulation of T cell subsets including Th9 cells. Interestingly, miR-24 and miR-27 clusters collaboratively suppress Th2 responses via targeting IL-4 and GATA3, respectively (104). Renz group developed the Impact of Differential Expression Across Layers (IDEAL) methodology, that prioritized miRNAs targeting in disease module, then identified that 5 top miRNAs including miR-23b, miR-27b, miR-106b, miR-203, and miR-206 in CD4^+^ Th2 cells (107). Especially, miR-23b and miR-27b indicate maximal impact for the stability of Th2 cells. These two miRNAs have biological roles for regulation of TGF/Smad2/3, EGFR1 and Th2-specific cytokine pathways such as IL-5, IL-9, IL-13, as well as GATA3 (107). These findings offer potential novel therapeutic interventions for asthma and other Th2-driven diseases.

It has been known that Th2 cells could be converted into Th9 cells in response to TGF-β (108), and there have been many reports on the shared signaling mechanisms between Th2 and Th9 cells. Similar to Th2 cells, Th9 cells also rely on IL-4 and GATA3 expression for IL-9 production within the context of TGF-β/Smad signaling pathway (86). Therefore, by altering both Smad signaling and Th2-associated transcriptional pathways, the miR-23-27–24 cluster may indirectly influence Th9 differentiation within the broader TGF-β/IL-4 transcriptional landscape.

### 6.6 miRNA-493-5p

miR-493-5p has been identified to serve as a tumor suppressor role and prohibit the proliferation, invasion and migration of lung cancer (109), gastric cancer (110), liver cancer (111, 112) and breast cancer (113). Recently, one published study reported that DC-derived exosomal miR-493-5p could negatively regulate the expression of FOXO1 in allergic asthma, and then the differentiation of Th9 cells (114). The study indicated that miR-493-5p expression is significantly decreased in OVA-induced asthma mice model, while its mimic inhibits the expression of IL-9, IRF4, and FOXO1 (114). Additionally, because FOXO1 contributes to IL-9 transcription downstream of TGF-β and participates in Th9 effector maturation, miR-493-5p functions as an inhibitory regulator of the TGF-β-Th9 network, and its reduction in asthma creates a Th9-permissive environment.

### 6.7 miRNA-19b

miR-19b is included in miR17–92 cluster with other six miRNAs (miR-17, miR-18a, miR-19a, miR-20a, miR-91, and miR-92a) (115). Particularly, miR-19b is well known as an important key for the oncogenic properties of miR-17–92 cluster in immune cells including T cells. Among these miRNAs, miR-17 and miR-19b play a role to enhance Th1 differentiation and IFN-γ production, while to prevent inducible Treg differentiation (40). Meanwhile, miR-19b also promotes Th2 cytokines, especially IL-13 and IL-4, in human and mouse CD4^+^ T cells via direct targeting of phosphatase and tensin homolog (PTEN), SOCS1, and A20 in asthma (116). Excitingly, we have recently demonstrated that miR-19b is specifically induced by TGF-β/IL-4 stimulation, and its overexpression leads to increased IL-9 production *in vitro* (117). TGF-β/IL-4 signaling enhances NF-κB activation through the SMAD3-Ser (213)/TRAF6/TAK1 signaling cascade activation and leads to the upregulation of miR-19b (117). Notably, miR-19b promotes IL-9 gene expression and promotes Th9 differentiation by the downregulation of *E2f8* (117), which is a repressor for *Il9* gene transcription during Th9 differentiation (discussed below) (29). Among the miRNAs described, miR-19b provides one of the clearest examples of direct enhancement of the TGF-β-IL-9 axis, reinforcing its central role in Th9 lineage specification.

Together, these findings reveal that miRNAs regulate Th9 differentiation through two major mechanisms: (1) by modulating TGF-β-Smad2/3 signaling activation, thereby influencing the permissiveness of the TGF-β-Smad environment required for IL-9 induction; and (2) by targeting Th9-specific transcriptional regulators such as PU.1, IRF4, BATF, HIF-1α, HIF-2α and FOXO1. By integrating these pathways, the miRNA network fine-tunes both the initiation and magnitude of Th9 responses, positioning miRNAs as key modulators of Th9 biology across diverse physiological and pathological contexts.

**Figure 2:**
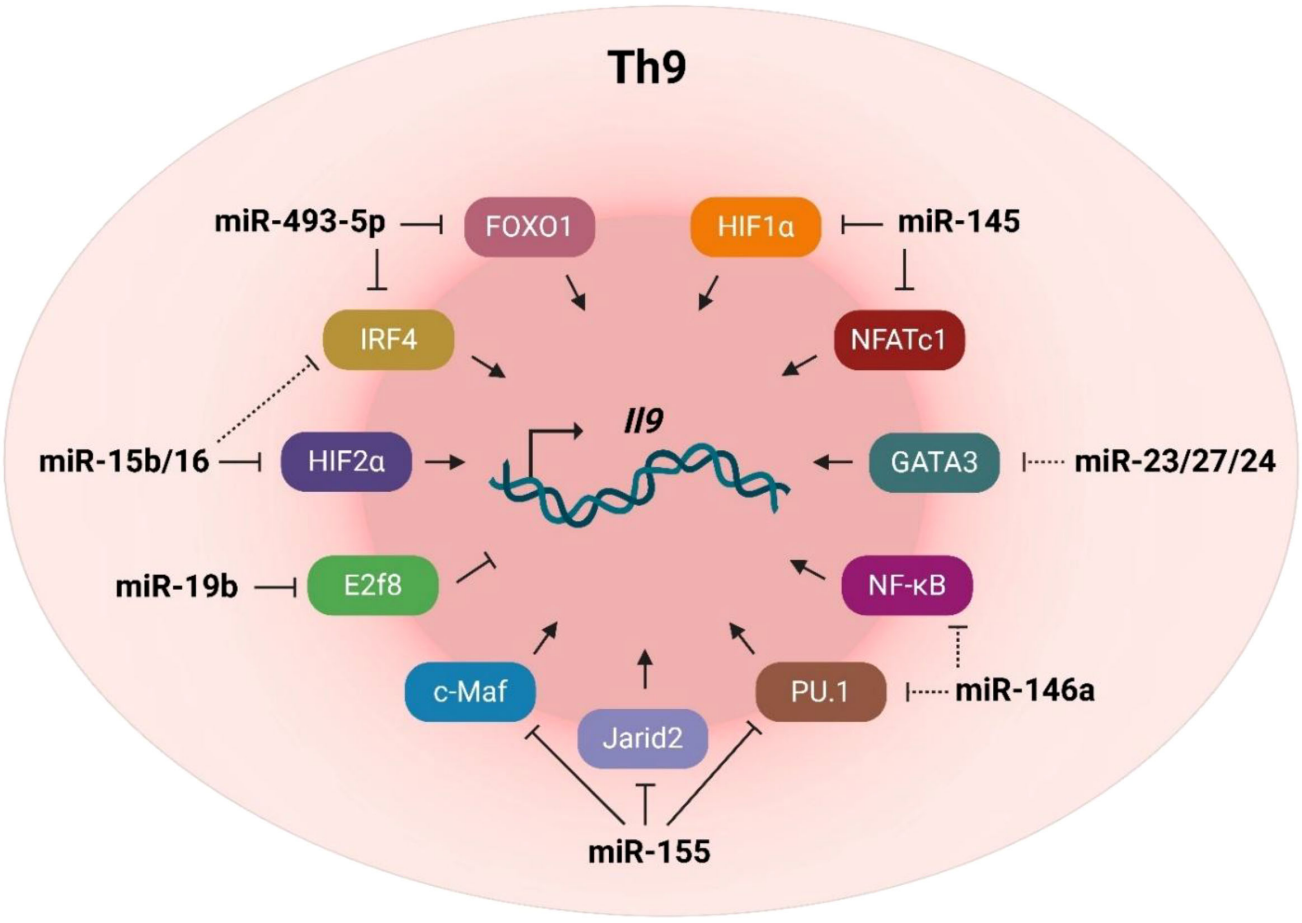
Overview of miRNAs in Th9 cells. This figure summarizes the best-established miRNAs associated with Th9 cells, and their targets that regulate *Il9* transcription.

We summarized that the signature cytokine of Th9 cells, IL-9, is regulated by several transcription factors targeted by unique miRNAs (**Figure 2**), which, in turn, can represent therapeutic targets for the treatment of various Th9-related diseases including cancers and autoimmune diseases.

## 7 miRNAs regulation of Th9 cells in diseases

Accumulated studies have investigated the role of miRNAs in the differentiation of Th subsets involved in the regulation of diseases pathogenesis (118, 119). Unlike other CD4^+^ T helper subsets, Th9 cells are functionally distinguished by their ability to produce IL-9 as a dominant effector cytokine, rather than being defined solely by a lineage-specific transcription factor (120, 121). This functional specialization enables Th9 cells to exert broad immunomodulatory effects across multiple cell types and disease contexts.

As mentioned above, miRNAs are widely recognized for their roles in regulating T cell activation and differentiation towards different subsets, as well as being considered as a marker and immunotherapy of human diseases (5, 119). Especially, in cancer, Th9 cells have been reported to play dual and opposing roles, exerting both anti-tumor and pro-tumor effects depending on the tumor microenvironment (10, 122–124). Meanwhile, Th9 cells represent potent Th effector cells involved in the pathogenesis of allergy/asthma (125, 126) and certain chronic inflammatory disorders (123). Given the growing evidence that miRNA regulatory networks in governing T cell responses are intimately involved in the pathogenesis of these diseases (127), it is not surprising that specific miRNAs play roles in regulating Th9 cell polarization and IL-9 production, consequently in the modulation of disease outcomes. Despite the limited number of studies, we highlight and summarize several representative examples to illustrate the current understanding in this regard.

### 7.1 Cancer

As mentioned above, Th9 cells exhibit dual functions in cancer immunology, functioning as potent anti-tumor effectors in most solid malignancies such as melanoma, colon cancer and breast cancer (10, 128, 129) while promoting tumor progression in certain hematologic malignancies (10, 122–124).

Beyond miRNA-mediated regulation, Th9 cells exert both anti-tumor and pro-tumor functions through IL-9-dependent and context-specific mechanisms (10, 123). In solid tumors, Th9 cells enhance anti-tumor immunity by promoting the recruitment and activation of cytotoxic CD8^+^ T cells and mast cells, as well as by improving antigen presentation through dendritic cell activation (128, 130). IL-9 can also directly influence tumor-associated immune responses by modulating the tumor microenvironment (130).

Conversely, Th9 cells may facilitate tumor progression in hematologic cancers (130). IL-9 can promote tumor cell survival and expansion, particularly in lymphoid malignancies, and can contribute to the formation of immunosuppressive microenvironments that dampen anti-tumor immunity (10, 123, 130). These divergent functions underscore the high degree of context-dependency that characterizes Th9 biology, providing essential mechanistic background for understanding how disease specific miRNA signatures further modulate Th9 function in cancer.

Thus, elucidating the expression patterns and functional roles of miRNAs in Th9 cells could be crucial for advancing our understanding of tumor development and the mechanisms underlying anti-tumor immunity. To begin with, miR-17–92 cluster including miR-19b has been shown to exert anti-tumorigenic properties by targeting the tumor-suppressor protein PTEN (40). However, it was unknown whether miR-19b plays a role in the differentiation of Th9 cells. Although there are also conflicting claims about the role of miR-19b, it is significantly overexpressed in plasma and tissues of patients with breast cancer (131, 132), suggesting a potential link between miR-19b and the regulation of Th9 cell activity in cancers. Based on these findings, it can be envisioned that miR-19b may be implicated in regulating functions of Th9 cells within the tumor microenvironment.

Despite the aforementioned findings, Th9 can also facilitate the progression of hematological tumors, where it exerts a tumorigenic role (124, 128). Although only partially understood, Th9 cells are known to have pro-tumor functions mediated by several miRNAs. While miR-19b has been shown to promote Th9-mediated tumorigenic activity, other miRNAs may exert opposing, tumor-suppressive effects on Th9 function and cancer progression. Among these, miR-21 is not only related to T cell differentiation through TGF-β signaling, but has also been extensively studied for its well-established tumor-promoting properties in cancers (133). Specifically, miR-21 can accelerate tumor growth by reduced activation of CD4^+^ and CD8^+^ T cells (134), largely due to its anti-apoptotic properties (135). Importantly, in acute myeloid leukemia (AML) patients, the miR-21 expression highly increases the expression of key Th9 cytokine, IL-9 inducing pro-tumoral response in T lymphocytes (136). Collectively, these findings strongly suggest that targeting miR-21 can restore tumor burden induced by activated Th9 cells.

miR-145 primarily functions as a tumor suppressor in many types of cancer (137). The expression of miR-145 in several cancers regulates tumor growth, invasion and metastasis through regulation of various signaling pathways. However, Th9 differentiation mediated by miR-145 still remains less well explored in various types of cancers. As mentioned earlier, this miRNA plays a critical role in inhibiting IL-9-producing Th9 cell differentiation though the HIF-1α signaling, with a significant negative correlation observed between miR-145 and Th9 cells in malignant ascites (MA) from liver cancer (74). According to this study, Th9 cells act as pro-tumor effector cells in MA, suggesting that their inhibition via miR-145 could be a potential therapeutic strategy. miR-200a has been also reported to attenuate the growth and metastasis of tumors by targeting such as β-catenin on pancreatic cancer cells (138). This study revealed that cancer cells actively suppress the expression of miR-200a using secreted IL-9 in tumor microenvironment (138). Although direct evidence demonstrating IL-9 production by Th9 cells under this condition is lacking, it is plausible that Th9 cells might contribute to the induction of IL-9, thereby potentially facilitating tumor-promoting activities though the suppression of miR-200a via an as-yet-unknown mechanism.

Taken together, these findings underscore the critical role of unique miRNAs that are either expressed in or regulate Th9 in modulating immune response against tumors. Consequently, therapeutic strategies targeting miRNAs hold significant promises as innovative approaches for cancer treatment. However, further investigation is required to elucidate the mechanism underlying various miRNAs expression and IL-9 inhibition on Th9 cells, as well as to determine how it directly affects the survival and migration of cancer cells within the tumor microenvironments. Given that the functional role of Th9 cells may differ depending on cancer type and progression, it is essential to develop more precise and context-dependent approaches when employing miRNA-based strategies to achieve effective anti-tumor responses.

### 7.2 Allergy

Th9 cells are key mediators of allergic asthma and other chronic inflammatory disorders (125, 126). In allergic asthma, Th9 cells contribute to disease pathogenesis primarily through IL-9-mediated effects on target cells (139, 140). IL-9 promotes the expansion and activation of mast cells, which release histamine and other inflammatory mediators that drive airway hyperresponsiveness (141). In addition, IL-9 enhances mucus production by airway epithelial cells, contributing to airway obstruction (140). Th9-derived IL-9 also facilitates eosinophil recruitment and survival, further amplifying type 2 inflammatory responses (142). These mechanisms collectively highlight the central role of Th9 cells in orchestrating allergic inflammation (139, 140). In the Th9-related diseases, high-throughput sequencing in PBMCs isolated from asthmatic children demonstrated differences in the expression of circulating miRNAs, among which miR-493-5p was selected due to substantial difference (114). The finding showed that miR-493-5p is downregulated in allergic asthma, which is related to increased Th9 cell differentiation. Some reports have also shown that the role of miR-143/145 in inhibitory regulation of Th9 by NFATc1 in asthma patients (75, 143). It suggests that miR-493-5p and miR-143/145 may play an important role in controlling Th9-related immunopathology, and thus may serve as a potential therapeutic target in asthma.

### 7.3 Autoimmune diseases

It has been known that miRNAs are important to maintain a proper balance between Th subsets from the immune system during autoimmune diseases (118, 144–146). miRNAs also regulate Th9 cells in autoimmune conditions. Notably, miR-15/16 has been identified as a critical suppressor of Tregs (91), as well as, Th17 cells (92), playing a key role in their pathogenesis. Simultaneously, miR-15b/16 has also been reported to indirectly reduce Th9 cells, thereby leading to a decrease in inflammatory responses of adoptive T cell transfer colitis model (93). At present, accumulating evidence suggests that miR-15b/16 is involved in the regulatory pathways within various T cell subsets under autoimmune conditions, indicating their potential as biomarkers for the clinical diagnosis of autoimmune diseases. To this end, further investigations are needed to elucidate the detailed regulatory mechanisms of miRNAs and their targets in Th cells including Th9.

miR-19b is another key regulator of Th9-mediated pathology in autoimmune diseases (117). We recently revealed that miR-19b expression is also upregulated in peripheral CD4^+^ T cells isolated from systemic sclerosis (SSc) patients, and the pathogenesis of SSc is attributed to miR-19-mediated Th9 cells (117). The expression of miR-19b in Th9 leads to exacerbated pathogenesis in SSc mice model, as well as in patients due to IL-9 production (117). Importantly, miR-19b inhibition significantly suppresses fibrogenesis and myofibroblast differentiation in the SSc mice model, which is due to reduced circulating IL-9 (117). Although this study is limited to the use of only SSc model, this model is acknowledged as ideal for investigating the function of miR-19b in Th9 cells. However, further investigations in other autoimmune disease models, including Inflammatory bowel disease (IBD), rheumatoid arthritis (RA) and EAE, are warranted. In line with this notion, miR-19b is significantly upregulated in MS patients compared to healthy donors (147, 148), although whether this is associated with Th9 cells remains unknown. Meanwhile, Guggino et al. (149) found that lower levels of miR-19b in γδ T cell from RA patients are correlated with the increased proinflammatory cytokine IL-6 production, suggesting a contribution of miR-19b to regulation of γδ T cell function and pathogenesis of RA. In Behçet’s uveitis (BU) disease, exosomal miR-19b absorbed by CD4^+^ T cells leads an imbalance in Treg/Th17 cells (decreasing the proportion of Tregs, but increasing the proportion of Th17 cells) by down-targeting CD46 (150). Given the significant role of miR-19b in regulating Th9 cells, it is interesting to further investigate its role in Th9 cells in these diseases.

### 7.4 Infectious and parasitic diseases

Th9 cells have been reported to be associated with the pathogenesis in bacterial infections. In infectious diseases, questions are being raised about the exact role of miRNAs in Th9 cells. The expression of miR-155 in Th9 cells was shown to be highly upregulated in BALF of MRSA patients, as well as in the lung tissues of MRSA-infected mice compared to the control group, and by targeting SIRT1 (103). In addition, Th9 differentiation by miR-155 during MRSA infection are positively correlated with the severity of the disease. These finding highlights miR-155 as evaluation indicators and as potential novel strategy for MRSA treatment.

In parasitic immunity, CD4^+^ T cells accumulate in local organs and skew towards Th2 and/or Tregs, which persist in parasites expulsion (151), and various miRNAs modulate important aspects of the parasite-host interaction play a crucial role in regulating parasites infection as immune cell-specific miRNA (152, 153). Remarkably, Th9 cells are also well-recognized for their critical role in defense against helminths and triggering host immune response (151, 154). It could be envisioned that certain miRNAs, including miR-155, miR-146a, and miR-21, which are closely associated with Th9 cells, might also influence differentiation and function of Th9 cell during parasitic infections (155).

Overall, miRNAs orchestrate Th9 cell differentiation and function across cancer, autoimmune, allergic and infectious diseases. They provide insights into disease mechanisms and represent promising targets for diagnostic and therapeutic strategies. Further investigations are needed to clarify precise molecular mechanisms, context-specific effects, and to develop miRNA-based approaches for Th9-related diseases. To provide a comprehensive overview of miRNA-mediated regulation of Th9 cells across different disease contexts, the key findings are summarized in **Table 3**.

**Table 3:** Summary of miRNAs regulating Th9 cells across different disease contexts.

Disease category	miRNA	Target(s)/pathway	Effect on Th9 cells	Functional outcome
Cancer	miR-19b	PTEN (putative)	Th9 ↑ (suggested)	Pro-/anti-tumor (Context-dependent)
	miR-21	TGF-β pathway, apoptosis genes	IL-9 production ↑	Tumor progression (AML)
	miR-145	HIF-1α	Th9 ↓	Tumor suppression (MA)
	miR-200a	β-catenin pathway (indirect via IL-9)	Th9 ↓	Tumor suppression
Allergy/Asthma	miR-493-5p	FOXO1	Th9 ↑ when miR ↓	Promotes allergic inflammation
	miR-143/145	NFATc1	Th9 ↓	Reduces asthma inflammation
Autoimmune diseases	miR-15b/16	HIF-2α	Th9 ↓	Reduces inflammation (Colitis)
	miR-19b	IL-9-associated signaling (E2f8↓)	Th9 ↑	Promotes fibrosis (SSc)
		IL-6 production	(Suggested involvement)	cytokine imbalance (RA)
		Treg/Th17 imbalance	(Suggested involvement)	dysregulation (BU)
Bacterial Infection	miR-155	SIRT1	Th9 ↑	Disease severity (MRSA)
Parasitic infections	miR-155	Immune regulatory pathways	(Suggested involvement)	Host defense modulation
	miR-146a	Immune regulatory pathways	(Suggested involvement)	Host defense modulation
	miR-21	TGF-β pathway	(Suggested involvement)	Host defense modulation

The table summarizes key miRNAs involved in Th9 cell differentiation and function, along with their molecular targets, effects on IL-9 production, and associated disease outcomes. Most findings are derived from pathological conditions, and in some cases, the effects on Th9 cells are inferred from related signaling pathways.

promotion (↑) or inhibition (↓) of Th9 cell differentiation of IL-9 production.

## 8 Conclusion and perspective

Mounting evidence highlights the pivotal role of miRNA-mediated pathways in the regulation of CD4^+^ T cells especially Th9 cells. As mentioned above, accumulated studies strongly support the hypothesis that miRNA targets are generally abundant in Th9 cells, suggesting that miRNAs regulate a broad spectrum of genes involved in the differentiation and function of Th9 cells. However, the regulatory mechanisms mediated by miRNAs with Th9 cells are still poorly characterized in Th9-associated diseases (12, 120). This is not only because the functional roles of Th9 cells in various disease contexts have not yet been clearly defined, but also studies investigating the interactions between Th9 cells and miRNAs under pathological conditions are still limited. Importantly, these limitations stem not merely from the lack of candidate miRNAs, but from insufficient integrative studies that connect non-coding RNA biology with context-specific immune signaling *in vivo* (5, 156). These gaps highlight the need for a deeper understanding of how Th9 cells contribute to immune regulation and disease pathogenesis through miRNA-associated mechanisms.

Up to now, most of these studies propose miRNAs as specific signatures for diagnosis and potential therapeutic targets in various diseases. Based on the identification and confirmation of miRNA functions as therapeutics, various strategies have been developed to find effective administration methods (157). Recent advances in miRNA-based therapeutics, including miRNA mimics, antagomirs, and exosome-mediated delivery systems, have provided promising approaches to modulate immune responses and potentially prevent or treat disease (158, 159). While exosome-mediated delivery systems show promise, the next frontier in Th9-related therapeutics involves the development of precision medicine tools, such as aptamer-conjugated nanoparticles or ligand-functionalized carriers, which enable the targeted delivery of miRNA modulators to specific T cell subsets while minimizing systemic off-target effects (160–163). In parallel, miRNA-based diagnostics for early disease detection have advanced, and currently, numerous diagnostic tools are already offered to clinicians (157, 164, 165). Furthermore, several clinical studies have explored the direct administration of miRNAs, highlighting their potential for durable immune modulation and disease prevention (166, 167).

Beyond their independent regulatory roles, emerging evidence suggests that miRNAs function within complex non-coding RNA networks. In particular, the interplay between long non-coding RNAs (lncRNAs) and miRNAs through competing endogenous RNA (ceRNA) mechanisms has added a new layer of post-transcriptional regulation within TGF-β signaling pathways (168). Given that TGF-β is indispensable for Th9 differentiation, the lncRNA-mediated sponging of miRNAs targeting key signaling intermediates, such as Smad3 and Th9-associated transcription factors, may critically shape lineage commitment and functional stability. This expanded regulatory framework suggests that Th9 differentiation is governed not only by canonical cytokine signaling and transcription factor networks, but also by finely tuned non-coding RNA crosstalk that integrates signal strength, timing, and cellular context.

Despite these advances, a coherent mechanistic model linking miRNA activity, ceRNA interactions, and activation-dependent TGF-β/Smad3 signaling in Th9 differentiation has yet to be fully established. The identification of novel miRNA targets and lncRNA-miRNA interactions within TGF-β/Smad signaling will be crucial for the development of refined therapeutic strategies against Th9-related diseases. Moreover, beyond lineage commitment, Th9 cells are increasingly appreciated as functionally plastic (9), and the miRNA-mediated epigenetic regulation is likely to contribute to the dynamic conversion between Th9 and other subsets, such as Th2 or Tregs, in response to the inflammatory milieu. In this context, understanding how non-coding RNA networks interface with signal-dependent transcriptional programs to stabilize or reprogram Th9 identity represents a critical unresolved question in helper T cell biology.

Further research is needed to dissect the regulatory networks of miRNAs in Th9 cells and to clarify their precise functions within various diseases environment. Furthermore, to enable the therapeutic application of miRNA candidates in Th9-related diseases, it will be essential to understand their detailed and specific functional characteristics. Ultimately, integrating miRNA-mediated regulation with ceRNA networks and activation-dependent Smad3 signaling will refine our conceptual framework of Th9 cell differentiation and accelerate the development of precision therapeutics for Th9-associated immune disorders.

## References

[1] Sun L Su Y Jiao A Wang X Zhang B . T cells in health and disease. Signal Transduc Tgt Ther. (2023) 8:235. doi: 10.1038/s41392-023-01471-y. PMID: 37332039 PMC10277291

[2] Fang D Zhu J . Dynamic balance between master transcription factors determines the fates and functions of CD4 T cell and innate lymphoid cell subsets. J Exp Med. (2017) 214:1861–76. doi: 10.1084/jem.20170494. PMID: 28630089 PMC5502437

[3] Jurgens AP Popovic B Wolkers MC . T cells at work: How post-transcriptional mechanisms control T cell homeostasis and activation. Eur J Immunol. (2021) 51:2178–87. doi: 10.1002/eji.202049055. PMID: 34180545 PMC8457102

[4] Rodriguez-Galan A Fernandez-Messina L Sanchez-Madrid F . Control of immunoregulatory molecules by miRNAs in T cell activation. Front Immunol. (2018) 9:2148. doi: 10.3389/fimmu.2018.02148, PMID: 30319616 PMC6167432

[5] Dosil SG Rodriguez-Galan A Sanchez-Madrid F Fernandez-Messina L . MicroRNAs in T cell-immunotherapy. Int J Mol Sci. (2022) 24(1):250. doi: 10.3390/ijms24010250. PMID: 36613706 PMC9820302

[6] Naqvi RA Datta M Khan SH Naqvi AR . Regulatory roles of microRNA in shaping T cell function, differentiation and polarization. Semin Cell Dev Biol. (2022) 124:34–47. doi: 10.1016/j.semcdb.2021.08.003. PMID: 34446356 PMC11661912

[7] Amado T Schmolka N Metwally H Silva-Santos B Gomes AQ . Cross-regulation between cytokine and microRNA pathways in T cells. Eur J Immunol. (2015) 45:1584–95. doi: 10.1002/eji.201545487. PMID: 25865116

[8] Podshivalova K Salomon DR . MicroRNA regulation of T-lymphocyte immunity: Modulation of molecular networks responsible for T-cell activation, differentiation, and development. Crit Rev Immunol. (2013) 33:435–76. doi: 10.1615/critrevimmunol.2013006858. PMID: 24099302 PMC4185288

[9] Liu X Li Y Wu W Huang H Hao H Song C . Regulatory mechanisms of Th9 cell differentiation. Front Immunol. (2025) 16:1650972. doi: 10.3389/fimmu.2025.1650972. PMID: 40948790 PMC12427788

[10] Chen T Guo J Cai Z Li B Sun L Shen Y . Th9 cell differentiation and its dual effects in tumor development. Front Immunol. (2020) 11:1026. doi: 10.3389/fimmu.2020.01026. PMID: 32508847 PMC7251969

[11] Chen W . TGF-beta regulation of T cells. Annu Rev Immunol. (2023) 41:483–512. doi: 10.1146/annurev-immunol-101921-045939. PMID: 36750317 PMC12453633

[12] Roostaee A Yaghobi R Afshari A Jafarinia M . Regulatory role of T helper 9/interleukin-9: Transplantation view. Heliyon. (2024) 10(4):e26359. doi: 10.1016/j.heliyon.2024.e26359. PMID: 38420400 PMC10900956

[13] O'Brien J Hayder H Zayed Y Peng C . Overview of microRNA biogenesis, mechanisms of actions, and circulation. Front Endocrinol Lausanne. (2018) 9:402. doi: 10.3389/fendo.2018.00402, PMID: 30123182 PMC6085463

[14] Shang R Lee S Senavirathne G Lai EC . MicroRNAs in action: Biogenesis, function and regulation. Nat Rev Genet. (2023) 24:816–33. doi: 10.1038/s41576-023-00611-y. PMID: 37380761 PMC11087887

[15] Ha M Kim VN . Regulation of microRNA biogenesis. Nat Rev Mol Cell Biol. (2014) 15:509–24. doi: 10.1038/nrm3838. PMID: 25027649

[16] Makarova J Turchinovich A Shkurnikov M Tonevitsky A . Extracellular miRNAs and cell-cell communication: Problems and prospects. Trends Biochem Sci. (2021) 46:640–51. doi: 10.1016/j.tibs.2021.01.007. PMID: 33610425

[17] Zhong X Heinicke F Rayner S . miRBaseMiner, a tool for investigating miRBase content. RNA Biol. (2019) 16:1534–46. doi: 10.1080/15476286.2019.1637680. PMID: 31251108 PMC6779376

[18] Filipowicz W Bhattacharyya SN Sonenberg N . Mechanisms of post-transcriptional regulation by microRNAs: Are the answers in sight? Nat Rev Genet. (2008) 9:102–14. doi: 10.1038/nrg2290. PMID: 18197166

[19] Forero A So L Savan R . Re-evaluating strategies to define the immunoregulatory roles of miRNAs. Trends Immunol. (2017) 38:558–66. doi: 10.1016/j.it.2017.06.001. PMID: 28666937 PMC5551433

[20] Jia Y Wei Y . Modulators of microRNA function in the immune system. Int J Mol Sci. (2020) 21(7):2357. doi: 10.3390/ijms21072357. PMID: 32235299 PMC7177468

[21] Xiao C Rajewsky K . MicroRNA control in the immune system: Basic principles. Cell. (2009) 136:26–36. doi: 10.1016/j.cell.2009.03.022. PMID: 19135886

[22] Vaghf A Khansarinejad B Ghaznavi-Rad E Mondanizadeh M . The role of microRNAs in diseases and related signaling pathways. Mol Biol Rep. (2022) 49:6789–801. doi: 10.1007/s11033-021-06725-y. PMID: 34718938

[23] Chen W Ten Dijke P . Immunoregulation by members of the TGFbeta superfamily. Nat Rev Immunol. (2016) 16:723–40. doi: 10.1038/nri.2016.112. PMID: 27885276

[24] Suzuki HI . MicroRNA control of TGF-beta signaling. Int J Mol Sci. (2018) 19(7):1901. doi: 10.3390/ijms19071901. PMID: 29958433 PMC6073626

[25] Wang A Pan D Lee YH Martinez GJ Feng XH Dong C . Cutting edge: Smad2 and Smad4 regulate TGF-beta-mediated Il9 gene expression via EZH2 displacement. J Immunol. (2013) 191:4908–12. doi: 10.4049/jimmunol.1300433. PMID: 24108699 PMC3842015

[26] Zhang YE . Non-Smad pathways in TGF-beta signaling. Cell Res. (2009) 19:128–39. doi: 10.1038/cr.2008.328. PMID: 19114990 PMC2635127

[27] Yan X Liu Z Chen Y . Regulation of TGF-beta signaling by smad7. Acta Biochim Biophys Sin Shanghai. (2009) 41:263–72. doi: 10.1093/abbs/gmp018. PMID: 19352540 PMC7110000

[28] Derynck R Zhang YE . Smad-dependent and Smad-independent pathways in TGF-beta family signalling. Nature. (2003) 425:577–84. doi: 10.1038/nature02006. PMID: 14534577

[29] Park SA Lim YJ Ku W Zhang D Cui K Tang LY . Opposing functions of circadian protein DBP and atypical E2F family E2F8 in anti-tumor Th9 cell differentiation. Nat Commun. (2022) 13:6069. doi: 10.1038/s41467-022-33733-8. PMID: 36241625 PMC9568563

[30] Diener C Hart M Kehl T Rheinheimer S Ludwig N Krammes L . Quantitative and time-resolved miRNA pattern of early human T cell activation. Nucleic Acids Res. (2020) 48:10164–83. doi: 10.1093/nar/gkaa788. PMID: 32990751 PMC7544210

[31] Liu S Iorgulescu JB Li S Borji M Barrera-Lopez IA Shanmugam V . Spatial maps of T cell receptors and transcriptomes reveal distinct immune niches and interactions in the adaptive immune response. Immunity. (2022) 55:1940–52. doi: 10.1016/j.immuni.2022.09.002. PMID: 36223726 PMC9745674

[32] Jurberg AD Vasconcelos-Fontes L Cotta-de-Almeida V . A tale from TGF-beta superfamily for thymus ontogeny and function. Front Immunol. (2015) 6:442. doi: 10.3389/fimmu.2015.00442. PMID: 26441956 PMC4564722

[33] Salmond RJ . Regulation of T cell activation and metabolism by transforming growth factor-beta. Biol Bsl. (2023) 12(2):297. doi: 10.3390/biology12020297. PMID: 36829573 PMC9953227

[34] An Q Duan L Wang Y Wang F Liu X Liu C . Role of CD4(+) T cells in cancer immunity: A single-cell sequencing exploration of tumor microenvironment. J Transl Med. (2025) 23:179. doi: 10.1186/s12967-025-06167-1, PMID: 39953548 PMC11829416

[35] Massague J Sheppard D . TGF-beta signaling in health and disease. Cell. (2023) 186:4007–37. doi: 10.1016/j.cell.2023.07.036, PMID: 37714133 PMC10772989

[36] Laudisi F Stolfi C Monteleone I Monteleone G . TGF-beta1 signaling and Smad7 control T-cell responses in health and immune-mediated disorders. Eur J Immunol. (2023) 53:e2350460. doi: 10.1002/eji.202350460. PMID: 37611637

[37] Dahmani A Janelle V Carli C Richaud M Lamarche C Khalili M . TGFbeta programs central memory differentiation in ex vivo-stimulated human T cells. Cancer Immunol Res. (2019) 7:1426–39. doi: 10.1158/2326-6066.cir-18-0691. PMID: 31308016

[38] Severin ME Lee PW Liu Y Selhorst AJ Gormley MG Pei W . MicroRNAs targeting TGFbeta signalling underlie the regulatory T cell defect in multiple sclerosis. Brain. (2016) 139:1747–61. doi: 10.1093/brain/aww084. PMID: 27190026 PMC4892757

[39] Kimura K Hohjoh H Yamamura T . The role for exosomal microRNAs in disruption of regulatory T cell homeostasis in multiple sclerosis. J Exp Neurosci. (2018) 12:1179069518764892. doi: 10.1177/1179069518764892. PMID: 29623002 PMC5881976

[40] Jiang S Li C Olive V Lykken E Feng F Sevilla J . Molecular dissection of the miR-17–92 cluster's critical dual roles in promoting Th1 responses and preventing inducible Treg differentiation. Blood. (2011) 118:5487–97. doi: 10.1182/blood-2011-05-355644. PMID: 21972292 PMC3217351

[41] Meira M Sievers C Hoffmann F Rasenack M Kuhle J Derfuss T . Unraveling natalizumab effects on deregulated miR-17 expression in CD4+ T cells of patients with relapsing-remitting multiple sclerosis. J Immunol Res. (2014) 2014:897249. doi: 10.1155/2014/897249. PMID: 24901013 PMC4036714

[42] Talebi F Ghorbani S Chan WF Boghozian R Masoumi F Ghasemi S . MicroRNA-142 regulates inflammation and T cell differentiation in an animal model of multiple sclerosis. J Neuroinflamm. (2017) 14:55. doi: 10.1186/s12974-017-0832-7. PMID: 28302134 PMC5356264

[43] Gauthy E Cuende J Stockis J Huygens C Lethe B Collet JF . GARP is regulated by miRNAs and controls latent TGF-beta1 production by human regulatory T cells. PloS One. (2013) 8:e76186. doi: 10.1371/journal.pone.0076186. PMID: 24098777 PMC3787020

[44] Zhou QH Haupt S Prots I Thümmler K Kremmer E Lipsky PE . miR-142-3p is involved in CD25^+^ CD4 T cell proliferation by targeting the expression of glycoprotein A repetitions predominant. J Immunol. (2013) 190:6579–88. doi: 10.4049/jimmunol.1202993. PMID: 23650616

[45] Gregory PA Bracken CP Smith E Bert AG Wright JA Roslan S . An autocrine TGF-beta/ZEB/miR-200 signaling network regulates establishment and maintenance of epithelial-mesenchymal transition. Mol Biol Cell. (2011) 22:1686–98. doi: 10.1091/mbc.e11-02-0103. PMID: 21411626 PMC3093321

[46] Chu JYS Chau MKM Chan CCY Tai ACP Cheung KF Chan TM . miR-200c prevents TGF-beta1-induced epithelial-to-mesenchymal transition and fibrogenesis in mesothelial cells by targeting ZEB2 and Notch1. Mol Ther Nucleic Acids. (2019) 17:78–91. doi: 10.1016/j.omtn.2019.05.008. PMID: 31226520 PMC6586597

[47] Erkeland SJ Stavast CJ Schilperoord-Vermeulen J Dal Collo G Van de Werken HJG Leon LG . The miR-200c/141-ZEB2-TGFbeta axis is aberrant in human T-cell prolymphocytic leukemia. Haematologica. (2022) 107:143–53. doi: 10.3324/haematol.2020.263756. PMID: 33596640 PMC8719092

[48] Rau CN Severin ME Lee PW Deffenbaugh JL Liu Y Murphy SP . MicroRNAs targeting TGF-beta signaling exacerbate central nervous system autoimmunity by disrupting regulatory T cell development and function. Eur J Immunol. (2024) 54(6):e2350548. doi: 10.1002/eji.202350548. PMID: 38634287 PMC11156541

[49] Zidan M Zidan AA Attia Saad M El-Shanshory M Bakry U Sobh A . Altered microRNA expression profile is linked to T-cell exhaustion-related pathways in pediatric patients with acute lymphoblastic leukemia. Hum Immunol. (2023) 84:113–22. doi: 10.1016/j.humimm.2022.10.005. PMID: 36347735

[50] Wang Y Tu S Huang Y Qin K Chen Z . MicroRNA-181a regulates Treg functions via TGF-beta1/Smad axis in the spleen of mice with acute gouty arthritis induced by MSU crystals. Braz J Med Biol Res. (2022) 55:e12002. doi: 10.1590/1414-431x2022e12002. PMID: 36477951 PMC9728631

[51] Zhang Z Xue Z Liu Y Liu H Guo X Li Y . MicroRNA-181c promotes Th17 cell differentiation and mediates experimental autoimmune encephalomyelitis. Brain Behav Immun. (2018) 70:305–14. doi: 10.1016/j.bbi.2018.03.011. PMID: 29545117

[52] Murugaiyan G da Cunha AP Ajay AK Joller N Garo LP Kumaradevan S . MicroRNA-21 promotes Th17 differentiation and mediates experimental autoimmune encephalomyelitis. J Clin Invest. (2015) 125:1069–80. doi: 10.1172/jci74347. PMID: 25642768 PMC4362225

[53] Li S Fan Q He S Tang T Liao Y Xie J . MicroRNA-21 negatively regulates Treg cells through a TGF-beta1/Smad-independent pathway in patients with coronary heart disease. Cell Physiol Biochem. (2015) 37:866–78. doi: 10.1159/000430214. PMID: 26383248

[54] Ando Y Yang GX Kenny TP Kawata K Zhang W Huang W . Overexpression of microRNA-21 is associated with elevated pro-inflammatory cytokines in dominant-negative TGF-beta receptor type II mouse. J Autoimmun. (2013) 41:111–9. doi: 10.1016/j.jaut.2012.12.013. PMID: 23395552 PMC3622842

[55] Soheilifar MH Vaseghi H Seif F Ariana M Ghorbanifar S Habibi N . Concomitant overexpression of mir-182-5p and mir-182-3p raises the possibility of IL-17-producing Treg formation in breast cancer by targeting CD3d, ITK, FOXO1, and NFATs: A meta-analysis and experimental study. Cancer Sci. (2021) 112:589–603. doi: 10.1111/cas.14764. PMID: 33283362 PMC7893989

[56] Qin A Wen Z Zhou Y Li Y Li Y Luo J . MicroRNA-126 regulates the induction and function of CD4(+) Foxp3(+) regulatory T cells through PI3K/AKT pathway. J Cell Mol Med. (2013) 17:252–64. doi: 10.1111/jcmm.12003, PMID: 23301798 PMC3822588

[57] Zhou Y Li Y Lu J Hong X Xu L . MicroRNA-30a controls the instability of inducible CD4+ Tregs through SOCS1. Mol Med Rep. (2019) 20:4303–14. doi: 10.3892/mmr.2019.10666. PMID: 31545427

[58] Li JQ . miR-106b-5p induces immune imbalance of Treg/Th17 in immune thrombocytopenic purpura through NR4A3/Foxp3 pathway. Cell Cycle. (2020) 19:1265–74. doi: 10.1080/15384101.2020.1746485. PMID: 32323598 PMC7469554

[59] Wang XY Chen XY Li J Zhang HY Liu J Sun LD . MiR-200a expression in CD4+ T cells correlates with the expression of Th17/Treg cells and relevant cytokines in psoriasis vulgaris: A case control study. BioMed Pharmacother. (2017) 93:1158–64. doi: 10.1016/j.biopha.2017.06.055. PMID: 28738533

[60] Becker W Nagarkatti M Nagarkatti PS . miR-466a targeting of TGF-beta2 contributes to foxP3(+) regulatory T cell differentiation in a murine model of allogeneic transplantation. Front Immunol. (2018) 9:688. doi: 10.3389/fimmu.2018.00688. PMID: 29686677 PMC5900016

[61] Takahashi H Kanno T Nakayamada S Hirahara K Sciume G Muljo SA . TGF-beta and retinoic acid induce the microRNA miR-10a, which targets Bcl-6 and constrains the plasticity of helper T cells. Nat Immunol. (2012) 13:587–95. doi: 10.1038/ni.2286. PMID: 22544395 PMC3499969

[62] Bronevetsky Y Burt TD McCune JM . Lin28b regulates fetal regulatory T cell differentiation through modulation of TGF-beta signaling. J Immunol. (2016) 197:4344–50. doi: 10.4049/jimmunol.1601070. PMID: 27793996 PMC5123822

[63] Das LM Torres-Castillo MD Gill T Levine AD . TGF-beta conditions intestinal T cells to express increased levels of miR-155, associated with down-regulation of IL-2 and itk mRNA. Mucosal Immunol. (2013) 6:167–76. doi: 10.1038/mi.2012.60. PMID: 22785227 PMC3504619

[64] Gordino G Costa-Pereira S Corredeira P Alves P Costa L Gomes AQ . MicroRNA-181a restricts human gammadelta T cell differentiation by targeting Map3k2 and Notch2. EMBO Rep. (2022) 23:e52234. doi: 10.15252/embr.202052234. PMID: 34821000 PMC8728617

[65] Wu R Zeng J Yuan J Deng X Huang Y Chen L . MicroRNA-210 overexpression promotes psoriasis-like inflammation by inducing Th1 and Th17 cell differentiation. J Clin Invest. (2018) 128:2551–68. doi: 10.1172/jci97426. PMID: 29757188 PMC5983326

[66] Gu Y Zhou H Yu H Yang W Wang B Qian F . miR-99a regulates CD4(+) T cell differentiation and attenuates experimental autoimmune encephalomyelitis by mTOR-mediated glycolysis. Mol Ther Nucleic Acids. (2021) 26:1173–85. doi: 10.1016/j.omtn.2021.07.010. PMID: 34820151 PMC8598972

[67] Ichiyama K Gonzalez-Martin A Kim BS Jin HY Jin W Xu W . The microRNA-183-96–182 cluster promotes T helper 17 cell pathogenicity by negatively regulating transcription factor foxo1 expression. Immunity. (2016) 44:1284–98. doi: 10.1016/j.immuni.2016.05.015. PMID: 27332731 PMC4918454

[68] Mikami Y Philips RL Sciume G Petermann F Meylan F Nagashima H . MicroRNA-221 and -222 modulate intestinal inflammatory Th17 cell response as negative feedback regulators downstream of interleukin-23. Immunity. (2021) 54:514–25:e516. doi: 10.1016/j.immuni.2021.02.015. PMID: 33657395 PMC8025838

[69] Wong MT Ye JJ Alonso MN Landrigan A Cheung RK Engleman E . Regulation of human Th9 differentiation by type I interferons and IL-21. Immunol Cell Biol. (2010) 88:624–31. doi: 10.1038/icb.2010.53. PMID: 20421880 PMC3090036

[70] Beriou G Bradshaw EM Lozano E Costantino CM Hastings WD Orban T . TGF-beta induces IL-9 production from human Th17 cells. J Immunol. (2010) 185:46–54. doi: 10.4049/jimmunol.1000356. PMID: 20498357 PMC2936106

[71] Xue G Jin G Fang J Lu Y . IL-4 together with IL-1beta induces antitumor Th9 cell differentiation in the absence of TGF-beta signaling. Nat Commun. (2019) 10:1376. doi: 10.1038/s41467-019-09401-9. PMID: 30914642 PMC6435687

[72] Dardalhon V Awasthi A Kwon H Galileos G Gao W Sobel RA . IL-4 inhibits TGF-beta-induced Foxp3+ T cells and, together with TGF-beta, generates IL-9+ IL-10+ Foxp3(-) effector T cells. Nat Immunol. (2008) 9:1347–55. doi: 10.1038/ni.1677. PMID: 18997793 PMC2999006

[73] Nakatsukasa H Zhang D Maruyama T Chen H Cui K Ishikawa M . The DNA-binding inhibitor Id3 regulates IL-9 production in CD4(+) T cells. Nat Immunol. (2015) 16:1077–84. doi: 10.1038/ni.3252. PMID: 26322481 PMC5935106

[74] Huang YY Jiang HX Shi QY Qiu X Wei X Zhang XL . miR-145 inhibits th9 cell differentiation by suppressing activation of the PI3K/akt/mTOR/p70S6K/HIF-1alpha pathway in Malignant ascites from liver cancer. Onco Targets Ther. (2020) 13:3789–800. doi: 10.2147/ott.s245346. PMID: 32440147 PMC7211301

[75] Qiu X Shi Q Huang Y Jiang H Qin S . miR-143/145 inhibits Th9 cell differentiation by targeting NFATc1. Mol Immunol. (2021) 132:184–91. doi: 10.1016/j.molimm.2021.01.001. PMID: 33446394

[76] Hermann-Kleiter N Baier G . NFAT pulls the strings during CD4+ T helper cell effector functions. Blood. (2010) 115:2989–97. doi: 10.1182/blood-2009-10-233585. PMID: 20103781

[77] Macian F . NFAT proteins: key regulators of T-cell development and function. Nat Rev Immunol. (2005) 5:472–84. doi: 10.1038/nri1632. PMID: 15928679

[78] Jash A Sahoo A Kim GC Chae CS Hwang JS Kim JE . Nuclear factor of activated T cells 1 (NFAT1)-induced permissive chromatin modification facilitates nuclear factor-kappaB (NF-kappaB)-mediated interleukin-9 (IL-9) transactivation. J Biol Chem. (2012) 287:15445–57. doi: 10.1074/jbc.m112.340356. PMID: 22427656 PMC3346086

[79] Wang S Zhang X Ju Y Zhao B Yan X Hu J . MicroRNA-146a feedback suppresses T cell immune function by targeting Stat1 in patients with chronic hepatitis B. J Immunol. (2013) 191:293–301. doi: 10.4049/jimmunol.1202100. PMID: 23698745

[80] Mohnle P Schutz SV van der Heide V Hubner M Luchting B Sedlbauer J . MicroRNA-146a controls Th1-cell differentiation of human CD4+ T lymphocytes by targeting PRKCepsilon. Eur J Immunol. (2015) 45:260–72. doi: 10.1002/eji.201444744, PMID: 25308712

[81] Lu LF Boldin MP Chaudhry A Lin LL Taganov KD Hanada T . Function of miR-146a in controlling Treg cell-mediated regulation of Th1 responses. Cell. (2010) 142:914–29. doi: 10.1016/j.cell.2010.08.012. PMID: 20850013 PMC3049116

[82] Okoye IS Czieso S Ktistaki E Roderick K Coomes SM Pelly VS . Transcriptomics identified a critical role for Th2 cell-intrinsic miR-155 in mediating allergy and antihelminth immunity. Proc Natl Acad Sci USA. (2014) 111:E3081–90. doi: 10.1073/pnas.1406322111. PMID: 25024218 PMC4121777

[83] Jurkin J Schichl YM Koeffel R Bauer T Richter S Konradi S . miR-146a is differentially expressed by myeloid dendritic cell subsets and desensitizes cells to TLR2-dependent activation. J Immunol. (2010) 184:4955–65. doi: 10.4049/jimmunol.0903021. PMID: 20375304

[84] Ghorpade DS Sinha AY Holla S Singh V Balaji KN . NOD2-nitric oxide-responsive microRNA-146a activates Sonic hedgehog signaling to orchestrate inflammatory responses in murine model of inflammatory bowel disease. J Biol Chem. (2013) 288:33037–48. doi: 10.1074/jbc.m113.492496. PMID: 24092752 PMC3829153

[85] Atarod S Ahmed MM Lendrem C Pearce KF Cope W Norden J . miR-146a and miR-155 expression levels in acute graft-versus-host disease incidence. Front Immunol. (2016) 7:56. doi: 10.3389/fimmu.2016.00056. PMID: 27014257 PMC4782155

[86] Chen J Guan L Tang L Liu S Zhou Y Chen C . T helper 9 cells: A new player in immune-related diseases. DNA Cell Biol. (2019) 38:1040–7. doi: 10.1089/dna.2019.4729. PMID: 31414895 PMC6791470

[87] Yang L Boldin MP Yu Y Liu CS Ea CK Ramakrishnan P . miR-146a controls the resolution of T cell responses in mice. J Exp Med. (2012) 209:1655–70. doi: 10.1084/jem.20112218. PMID: 22891274 PMC3428948

[88] Gagnon JD Kageyama R Shehata HM Fassett MS Mar DJ Wigton EJ . miR-15/16 restrain memory T cell differentiation, cell cycle, and survival. Cell Rep. (2019) 28:2169–81:e2164. doi: 10.2139/ssrn.3280244 PMC6715152 31433990

[89] Hutter K Rulicke T Szabo TG Andersen L Villunger A Herzog S . The miR-15a/16–1 and miR-15b/16–2 clusters regulate early B cell development by limiting IL-7 receptor expression. Front Immunol. (2022) 13:967914. doi: 10.3389/fimmu.2022.967914. PMID: 36110849 PMC9469637

[90] Singh Y Garden OA Lang F Cobb BS . MicroRNA-15b/16 enhances the induction of regulatory T cells by regulating the expression of rictor and mTOR. J Immunol. (2015) 195:5667–77. doi: 10.4049/jimmunol.1401875. PMID: 26538392 PMC4671309

[91] Dong J Huth WJ Marcel N Zhang Z Lin LL Lu LF . miR-15/16 clusters restrict effector Treg cell differentiation and function. J Exp Med. (2023) 220(10):e20230321. doi: 10.1084/jem.20230321. PMID: 37516921 PMC10374942

[92] Liu R Ma X Chen L Yang Y Zeng Y Gao J . MicroRNA-15b suppresses th17 differentiation and is associated with pathogenesis of multiple sclerosis by targeting O-glcNAc transferase. J Immunol. (2017) 198:2626–39. doi: 10.4049/jimmunol.1601727. PMID: 28228555

[93] Singh Y Garden OA Lang F Cobb BS . MicroRNAs regulate T-cell production of interleukin-9 and identify hypoxia-inducible factor-2alpha as an important regulator of T helper 9 and regulatory T-cell differentiation. Immunology. (2016) 149:74–86. doi: 10.1111/imm.12631. PMID: 27278750 PMC4981607

[94] Chen L Gao D Shao Z Zheng Q Yu Q . miR-155 indicates the fate of CD4(+) T cells. Immunol Lett. (2020) 224:40–9. doi: 10.1016/j.imlet.2020.05.003. PMID: 32485191

[95] Escobar TM Kanellopoulou C Kugler DG Kilaru G Nguyen CK Nagarajan V . miR-155 activates cytokine gene expression in Th17 cells by regulating the DNA-binding protein Jarid2 to relieve polycomb-mediated repression. Immunity. (2014) 40:865–79. doi: 10.1016/j.immuni.2014.03.014. PMID: 24856900 PMC4092165

[96] Hu J Huang S Liu X Zhang Y Wei S Hu X . miR-155: an important role in inflammation response. J Immunol Res. (2022) 2022:7437281. doi: 10.1155/2022/7437281. PMID: 35434143 PMC9007653

[97] Rodriguez A Vigorito E Clare S Warren MV Couttet P Soond DR . Requirement of bic/microRNA-155 for normal immune function. Science. (2007) 316:608–11. doi: 10.1007/978-1-4419-1005-9_463 PMC2610435 17463290

[98] Kim HJ Park SO Byeon HW Eo JC Choi JY Tanveer M . T cell-intrinsic miR-155 is required for Th2 and Th17-biased responses in acute and chronic airway inflammation by targeting several different transcription factors. Immunology. (2022) 166:357–79. doi: 10.1111/imm.13477. PMID: 35404476

[99] Malmhall C Alawieh S Lu Y Sjostrand M Bossios A Eldh M . MicroRNA-155 is essential for T(H)2-mediated allergen-induced eosinophilic inflammation in the lung. J Allergy Clin Immunol. (2014) 133:1429–38:e1421-1427. doi: 10.1016/j.jaci.2013.11.008, PMID: 24373357

[100] Banerjee A Schambach F DeJong CS Hammond SM Reiner SL . Micro-RNA-155 inhibits IFN-gamma signaling in CD4+ T cells. Eur J Immunol. (2010) 40:225–31. doi: 10.1002/eji.200939381, PMID: 19877012 PMC2807623

[101] O'Connell RM Kahn D Gibson WS Round JL Scholz RL Chaudhuri AA . MicroRNA-155 promotes autoimmune inflammation by enhancing inflammatory T cell development. Immunity. (2010) 33:607–19. doi: 10.1016/j.immuni.2010.09.009, PMID: 20888269 PMC2966521

[102] Zhu F Li H Liu Y Tan C Liu X Fan H . miR-155 antagomir protect against DSS-induced colitis in mice through regulating Th17/Treg cell balance by Jarid2/Wnt/beta-catenin. BioMed Pharmacother. (2020) 126:109909. doi: 10.1016/j.biopha.2020.109909. PMID: 32135463

[103] Tian K Xu W . MiR-155 regulates Th9 differentiation in children with methicillin-resistant Staphylococcus aureus pneumonia by targeting SIRT1. Hum Immunol. (2021) 82:775–81. doi: 10.1016/j.humimm.2021.07.002. PMID: 34294459

[104] Cho S Wu CJ Yasuda T Cruz LO Khan AA Lin LL . miR-23 approximately 27 approximately 24 clusters control effector T cell differentiation and function. J Exp Med. (2016) 213:235–49. doi: 10.1083/jcb.2124oia22 PMC4749926 26834155

[105] Cruz LO Hashemifar SS Wu CJ Cho S Nguyen DT Lin LL . Excessive expression of miR-27 impairs Treg-mediated immunological tolerance. J Clin Invest. (2017) 127:530–42. doi: 10.1172/jci88415. PMID: 28067667 PMC5272185

[106] Rogler CE Levoci L Ader T Massimi A Tchaikovskaya T Norel R . MicroRNA-23b cluster microRNAs regulate transforming growth factor-beta/bone morphogenetic protein signaling and liver stem cell differentiation by targeting Smads. Hepatology. (2009) 50:575–84. doi: 10.1002/hep.22982. PMID: 19582816

[107] Kilic A Santolini M Nakano T Schiller M Teranishi M Gellert P . A systems immunology approach identifies the collective impact of 5 miRs in Th2 inflammation. JCI Insight. (2018) 3(11):e97503. doi: 10.1172/jci.insight.97503, PMID: 29875322 PMC6124409

[108] Veldhoen M Uyttenhove C van Snick J Helmby H Westendorf A Buer J . Transforming growth factor-beta 'reprograms' the differentiation of T helper 2 cells and promotes an interleukin 9-producing subset. Nat Immunol. (2008) 9:1341–6. doi: 10.1038/ni.1659. PMID: 18931678

[109] Gu Y Cheng Y Song Y Zhang Z Deng M Wang C . MicroRNA-493 suppresses tumor growth, invasion and metastasis of lung cancer by regulating E2F1. PloS One. (2014) 9:e102602. doi: 10.1371/journal.pone.0102602. PMID: 25105419 PMC4126682

[110] Zhou W Zhang C Jiang H Zhang Z Xie L He X . MiR-493 suppresses the proliferation and invasion of gastric cancer cells by targeting RhoC. Iran J Basic Med Sci. (2015) 18:1027–33. doi: 10.22038/ijbms.2015.6022 PMC4686574 26730339

[111] Wang G Fang X Han M Wang X Huang Q . MicroRNA-493-5p promotes apoptosis and suppresses proliferation and invasion in liver cancer cells by targeting VAMP2. Int J Mol Med. (2018) 41:1740–8. doi: 10.3892/ijmm.2018.3358. PMID: 29328362

[112] Yasukawa K Liew LC Hagiwara K Hironaka-Mitsuhashi A Qin XY Furutani Y . MicroRNA-493-5p-mediated repression of the MYCN oncogene inhibits hepatic cancer cell growth and invasion. Cancer Sci. (2020) 111:869–80. doi: 10.1111/cas.14292. PMID: 31883160 PMC7060481

[113] Zhao L Feng X Song X Zhou H Zhao Y Cheng L . miR-493-5p attenuates the invasiveness and tumorigenicity in human breast cancer by targeting FUT4. Oncol Rep. (2016) 36:1007–15. doi: 10.3892/or.2016.4882. PMID: 27375041

[114] Rao X Dong H Zhang W Sun H Gu W Zhang X . MiR-493-5p inhibits Th9 cell differentiation in allergic asthma by targeting FOXO1. Respir Res. (2022) 23:286. doi: 10.1186/s12931-022-02207-2. PMID: 36253857 PMC9578235

[115] Kuo G Wu CY Yang HY . MiR-17–92 cluster and immunity. J Formos Med Assoc. (2019) 118:2–6. doi: 10.1016/j.jfma.2018.04.013. PMID: 29857952

[116] Simpson LJ Patel S Bhakta NR Choy DF Brightbill HD Ren X . A microRNA upregulated in asthma airway T cells promotes TH2 cytokine production. Nat Immunol. (2014) 15:1162–70. doi: 10.1038/ni.3026. PMID: 25362490 PMC4233009

[117] Lim YJ Park SA Wang D Jin W Ku WL Zhang D . MicroRNA-19b exacerbates systemic sclerosis through promoting Th9 cells. Cell Rep. (2024) 43:114565. doi: 10.1016/j.celrep.2025.115447. PMID: 39083380 PMC11440512

[118] Sethi A Kulkarni N Sonar S Lal G . Role of miRNAs in CD4 T cell plasticity during inflammation and tolerance. Front Genet. (2013) 4:8. doi: 10.3389/fgene.2013.00008. PMID: 23386861 PMC3560369

[119] Baumjohann D Ansel KM . MicroRNA-mediated regulation of T helper cell differentiation and plasticity. Nat Rev Immunol. (2013) 13:666–78. doi: 10.1038/nri3494. PMID: 23907446 PMC3980848

[120] Xu WD Chen YY Li YW Yang J Huang AF . Targeting Th9 cells in autoimmune diseases: a narrative review. Front Immunol. (2025) 16:1615611. doi: 10.3389/fimmu.2025.1615611. PMID: 40771819 PMC12325201

[121] Zhao P Xiao X Ghobrial RM Li XC . IL-9 and Th9 cells: progress and challenges. Int Immunol. (2013) 25:547–51. doi: 10.1093/intimm/dxt039. PMID: 24027199 PMC3784066

[122] Kalim M Jing R Guo W Xing H Lu Y . Functional diversity and regulation of IL-9-producing T cells in cancer immunotherapy. Cancer Lett. (2024) 606:217306. doi: 10.1016/j.canlet.2024.217306. PMID: 39426662 PMC11675864

[123] Bick F Blanchetot C Lambrecht BN Schuijs MJ . A reappraisal of IL-9 in inflammation and cancer. Mucosal Immunol. (2025) 18:1–15. doi: 10.1016/j.mucimm.2024.10.003. PMID: 39389468

[124] Wan J Wu Y Ji X Huang L Cai W Su Z . IL-9 and IL-9-producing cells in tumor immunity. Cell Commun Signal. (2020) 18:50. doi: 10.1186/s12964-020-00538-5. PMID: 32228589 PMC7104514

[125] Soroosh P Doherty TA . Th9 and allergic disease. Immunology. (2009) 127:450–8. doi: 10.1111/j.1365-2567.2009.03114.x. PMID: 19604299 PMC2729522

[126] Angkasekwinai P . Th9 cells in allergic disease. Curr Allergy Asthma Rep. (2019) 19:29. doi: 10.1007/s11882-019-0860-8. PMID: 30915580

[127] Liu J Wu CP Lu BF Jiang JT . Mechanism of T cell regulation by microRNAs. Cancer Biol Med. (2013) 10:131–7. doi: 10.2174/1381612823666170714153424. PMID: 24379987 PMC3860337

[128] Rivera Vargas T Humblin E Vegran F Ghiringhelli F Apetoh L . T(H)9 cells in anti-tumor immunity. Semin Immunopathol. (2017) 39:39–46. doi: 10.1007/s00281-016-0599-4. PMID: 27832300 PMC5222918

[129] Benoit-Lizon I Apetoh L . Harnessing T(H)9 cells in cancer immunotherapy. Semin Immunol. (2021) 52:101477. doi: 10.1016/j.smim.2021.101477. PMID: 33893025

[130] Zheng N Lu Y . Targeting the IL-9 pathway in cancer immunotherapy. Hum Vaccin Immunother. (2020) 16:2333–40. doi: 10.1080/21645515.2019.1710413. PMID: 32040369 PMC7644168

[131] Zhao Q Shen L Lu J Xie H Li D Shang Y . A circulating miR-19b-based model in diagnosis of human breast cancer. Front Mol Biosci. (2022) 9:980841. doi: 10.3389/fmolb.2022.980841. PMID: 36188229 PMC9523242

[132] Li CS Zhang JW Ma ZL Zhang F Yu WL . miR-19b serves as a prognostic biomarker of breast cancer and promotes tumor progression through PI3K/AKT signaling pathway. Oncotargets Ther. (2018) 11:4087–95. doi: 10.2147/ott.s171043. PMID: 30038508 PMC6052917

[133] Smigielska-Czepiel K van den Berg A Jellema P Slezak-Prochazka I Maat H van den Bos H . Dual role of miR-21 in CD4+ T-cells: activation-induced miR-21 supports survival of memory T-cells and regulates CCR7 expression in naive T-cells. PloS One. (2013) 8:e76217. doi: 10.1371/journal.pone.0076217. PMID: 24098447 PMC3787993

[134] Chi LH Cross RSN Redvers RP Davis M Hediyeh-zadeh S Mathivanan S . MicroRNA-21 is immunosuppressive and pro-metastatic via separate mechanisms. Oncogenesis. (2022) 11(1):38. doi: 10.1038/s41389-022-00413-7. PMID: 35821197 PMC9276829

[135] Ruan Q Wang P Wang T Qi J Wei M Wang S . MicroRNA-21 regulates T-cell apoptosis by directly targeting the tumor suppressor gene Tipe2. Cell Death Dis. (2014) 5:e1095. doi: 10.1038/cddis.2014.47. PMID: 24577093 PMC3944261

[136] Moussa Agha D Rouas R Najar M Bouhtit F Fayyad-Kazan H Lagneaux L . Impact of bone marrow miR-21 expression on acute myeloid leukemia T lymphocyte fragility and dysfunction. Cells. (2020) 9(9):2053. doi: 10.3390/cells9092053. PMID: 32911844 PMC7563595

[137] Cui SY Wang R Chen LB . MicroRNA-145: a potent tumour suppressor that regulates multiple cellular pathways. J Cell Mol Med. (2014) 18:1913–26. doi: 10.1111/jcmm.12358. PMID: 25124875 PMC4244007

[138] Hu B Qiu-Lan H Lei RE Shi C Jiang HX Qin SY . Interleukin-9 Promotes Pancreatic Cancer Cells Proliferation and Migration via the miR-200a/Beta-Catenin Axis. BioMed Res Int. (2017) 2017:2831056. doi: 10.1155/2017/2831056. PMID: 28349057 PMC5352879

[139] Kaminuma O Kitamura N Gotoh M . Emerging role and function of th9 cells in allergic inflammation. J Inflammation Res. (2025) 18:16385–97. doi: 10.2147/jir.s546234. PMID: 41312549 PMC12649786

[140] Koch S Sopel N Finotto S . Th9 and other IL-9-producing cells in allergic asthma. Semin Immunopathol. (2017) 39:55–68. doi: 10.1007/s00281-016-0601-1. PMID: 27858144

[141] Sehra S Yao W Nguyen ET Glosson-Byers NL Akhtar N Zhou B . TH9 cells are required for tissue mast cell accumulation during allergic inflammation. J Allergy Clin Immunol. (2015) 136:433–40. doi: 10.1016/j.jaci.2015.01.021. PMID: 25746972 PMC4530056

[142] Lambrecht BN Ahmed E Hammad H . The immunology of asthma. Nat Immunol. (2025) 26:1233–45. doi: 10.1038/ni.3049. PMID: 40730897

[143] Koch S Graser A Mirzakhani H Zimmermann T Melichar VO Wolfel M . Increased expression of nuclear factor of activated T cells 1 drives IL-9-mediated allergic asthma. J Allergy Clin Immunol. (2016) 137:1898–902. doi: 10.1016/j.jaci.2015.11.047. PMID: 26993036 PMC4889777

[144] Scherm MG Daniel C . miRNA regulation of T cells in islet autoimmunity and type 1 diabetes. Curr Diabetes Rep. (2020) 20:41. doi: 10.1007/s11892-020-01325-9. PMID: 32725277 PMC7387371

[145] Soltanzadeh-Yamchi M Shahbazi M Aslani S Mohammadnia-Afrouzi M . MicroRNA signature of regulatory T cells in health and autoimmunity. BioMed Pharmacother. (2018) 100:316–23. doi: 10.1016/j.biopha.2018.02.030. PMID: 29453041

[146] Deng Y Wang Z Chang C Lu L Lau CS Lu Q . Th9 cells and IL-9 in autoimmune disorders: Pathogenesis and therapeutic potentials. Hum Immunol. (2017) 78:120–8. doi: 10.1016/j.humimm.2016.12.010. PMID: 28040536

[147] De Santis G Ferracin M Biondani A Caniatti L Rosaria Tola M Castellazzi M . Altered miRNA expression in T regulatory cells in course of multiple sclerosis. J Neuroimmunol. (2010) 226:165–71. doi: 10.1016/j.jneuroim.2010.06.009. PMID: 20637509

[148] Freiesleben S Hecker M Zettl UK Fuellen G Taher L . Analysis of microRNA and gene expression profiles in multiple sclerosis: integrating interaction data to uncover regulatory mechanisms. Sci Rep. (2016) 6:34512. doi: 10.1038/srep34512. PMID: 27694855 PMC5046091

[149] Guggino G Orlando V Saieva L Ruscitti P Cipriani P La Manna MP . Downregulation of miRNA17–92 cluster marks Vgamma9Vdelta2 T cells from patients with rheumatoid arthritis. Arthritis Res Ther. (2018) 20:236. doi: 10.1186/s13075-018-1740-7. PMID: 30348222 PMC6235230

[150] Jiang Q Wang Q Tan S Cai J Ye X Su G . Effects of plasma-derived exosomal miRNA-19b-3p on treg/T helper 17 cell imbalance in behcet's uveitis. Invest Ophthalmol Vis Sci. (2023) 64:28. doi: 10.1167/iovs.64.4.28. PMID: 37093132 PMC10148662

[151] Bouchery T Kyle R Ronchese F Le Gros G . The differentiation of CD4(+) t-helper cell subsets in the context of helminth parasite infection. Front Immunol. (2014) 5:487. doi: 10.3389/fimmu.2014.00487. PMID: 25360134 PMC4197778

[152] Rojas-Pirela M Andrade-Alviarez D Quinones W Rojas MV Castillo C Liempi A . MicroRNAs: critical players during helminth infections. Microorganisms. (2022) 11(1):61. doi: 10.3390/microorganisms11010061. PMID: 36677353 PMC9861972

[153] Ullah H Tian Y Arbab S Li K Khan MIU Rahman SU . Circulatory microRNAs in helminthiases: potent as diagnostics biomarker, its potential role and limitations. Front Vet Sci. (2022) 9:1018872. doi: 10.3389/fvets.2022.1018872. PMID: 36387413 PMC9650547

[154] Licona-Limon P Arias-Rojas A Olguin-Martinez E . Il-9 and th9 in parasite immunity. Semin Immunopathol. (2017) 39:29–38. doi: 10.1007/s00281-016-0606-9. PMID: 27900450

[155] Arora N Tripathi S Singh AK Mondal P Mishra A Prasad A . Micromanagement of immune system: role of miRNAs in helminthic infections. Front Microbiol. (2017) 8:586. doi: 10.3389/fmicb.2017.00586. PMID: 28450853 PMC5390025

[156] Altundas N Balkan E Kizilkaya M Demirci E . Exploring the role of miRNAs in hepatocellular carcinoma: insights into signaling pathways and their regulatory mechanisms. Pak J Med Sci. (2025) 41:1441–6. doi: 10.12669/pjms.41.5.11444. PMID: 40469123 PMC12130952

[157] Diener C Keller A Meese E . Emerging concepts of miRNA therapeutics: from cells to clinic. Trends Genet. (2022) 38:613–26. doi: 10.1016/j.tig.2022.02.006. PMID: 35303998

[158] Zhang Y Liu Q Zhang X Huang H Tang S Chai Y . Recent advances in exosome-mediated nucleic acid delivery for cancer therapy. J Nanobiotechnol. (2022) 20:279. doi: 10.1186/s12951-022-01472-z. PMID: 35701788 PMC9194774

[159] Lee M Lee M Song Y Kim S Park N . Recent advances and prospects of nucleic acid therapeutics for anti-cancer therapy. Molecules. (2024) 29(19):4737. doi: 10.3390/molecules29194737. PMID: 39407665 PMC11477775

[160] Driscoll J Gondaliya P Zinn DA Jain R Yan IK Dong H . Using aptamers for targeted delivery of RNA therapies. Mol Ther. (2025) 33:1344–67. doi: 10.1016/j.ymthe.2025.02.047. PMID: 40045577 PMC11997499

[161] Fu Z Xiang J . Aptamer-functionalized nanoparticles in targeted delivery and cancer therapy. Int J Mol Sci. (2020) 21(23):9123. doi: 10.3390/ijms21239123. PMID: 33266216 PMC7730239

[162] Esposito CL Catuogno S de Franciscis V . Aptamer-miRNA conjugates for cancer cell-targeted delivery. Methods Mol Biol. (2016) 1364:197–208. doi: 10.1007/978-1-4939-3112-5_16. PMID: 26472452

[163] Abdelaal AM Kasinski AL . Ligand-mediated delivery of RNAi-based therapeutics for the treatment of oncological diseases. NAR Cancer. (2021) 3:zcab030. doi: 10.1093/narcan/zcab030. PMID: 34316717 PMC8291076

[164] Ho PTB Clark IM Le LTT . Microrna-based diagnosis and therapy. Int J Mol Sci. (2022) 23(13):7167. doi: 10.3390/ijms23137167. PMID: 35806173 PMC9266664

[165] Sempere LF Azmi AS Moore A . Microrna-based diagnostic and therapeutic applications in cancer medicine. Wiley Interdiscip Rev RNA. (2021) 12:e1662. doi: 10.1002/wrna.1662. PMID: 33998154 PMC8519065

[166] Hanna J Hossain GS Kocerha J . The potential for microrna therapeutics and clinical research. Front Genet. (2019) 10:478. doi: 10.3389/fgene.2019.00478. PMID: 31156715 PMC6532434

[167] Iacomino G . Mirnas: the road from bench to bedside. Genes Bsl. (2023) 14(2):314. doi: 10.3390/genes14020314. PMID: 36833241 PMC9957002

[168] Lai L Wang Z Ge Y Qiu W Wu B Fang F . Comprehensive analysis of the long noncoding RNA-associated competitive endogenous RNA network in the osteogenic differentiation of periodontal ligament stem cells. BMC Genomics. (2022) 23:1. doi: 10.1186/s12864-021-08243-4. PMID: 34979896 PMC8725252

